# Supplementary material for: Characterizing sleep spindles in 11,630 individuals from the National Sleep Research Resource
Source: Nat Commun. 2017 Jun 26;8:15930. doi: 10.1038/ncomms15930 (PMC5490197; doi:10.1038/ncomms15930)
Supplement: Supplementary Information [file ncomms15930-s1.pdf]

**Title of file for HTML**: Supplementary Information
**Description**: Supplementary Figures, Supplementary Tables, Supplementary Note, Supplementary Methods and Supplementary References

**Title of file for HTML**: Peer Review File
Description:

# SUPPLEMENTARY INFORMATION

## SUPPLEMENTARY NOTE 1

This document contains the following sections:

- Supplementary Notes (page 1)
- Supplementary Figures (pages 2 – 31)
- Supplementary Tables (pages 32 – 64)
- Supplementary Methods (pages 65 – 66)
- Supplementary References (page 67)

Supplementary Figures and Tables below are grouped by topic:

| | | |
|---|---|---|
| Primary data | . | Figure S1 |
| Signal processing | Tables S1-S3 | Figures S2-S8 |
| Reliability of spindle measures | Table S4 | Figures S9 |
| Relation to sleep macro-architecture | Tables S5-S7 | . |
| Age-effects | Tables S8-S9 | Figures S10 |
| Fast/slow spindles | Table S10 | Figures S11-S13 |
| Within-night spindle dynamics | Tables S11, S12 | Figures S14-S19 |
| Clinical correlates | Tables S13-S16 | Figures S20 |
| Racial correlates | Tables S17-S22 | Figures S21-S23 |
| Heritability estimation | Tables S23-S26 | . |
| Topographical analyses | Table S27 | Figures S24, S25 |
| Alternative detection method | Tables S28-S29 | . |
| Threshold effects on spindle detection | Tables S30-S33 | Figures S26-S28 |

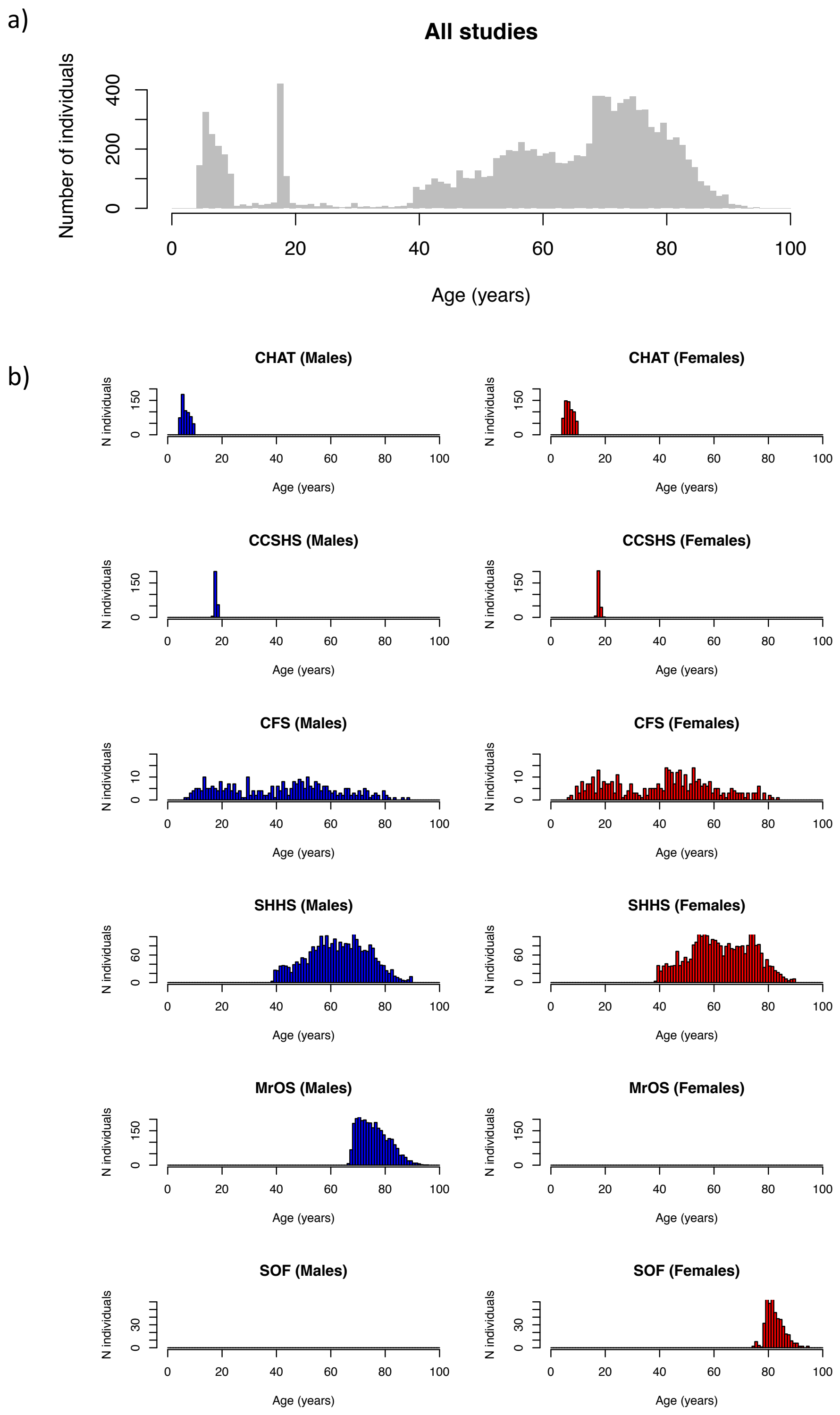


**Supplementary Figure 1. Age distribution in years, stratified by sex and study.** a) The age distribution across all six studies (age at baseline for studies with repeated measures). b) Age distributions stratified by study and sex.

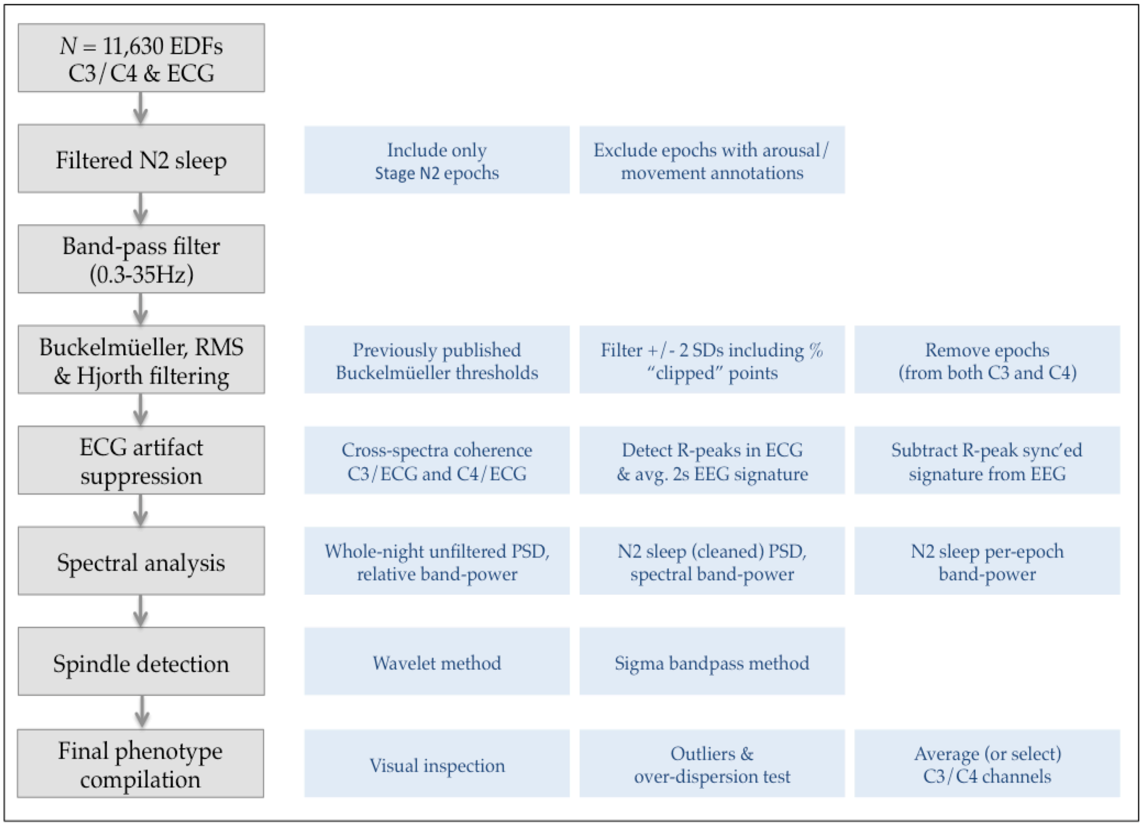


**Supplementary Figure 2. Schematic overview of the spindle detection pipeline.** In-house C/C++ software was developed to implement all steps in this pipeline (available from http://zzz.bwh.harvard.edu).

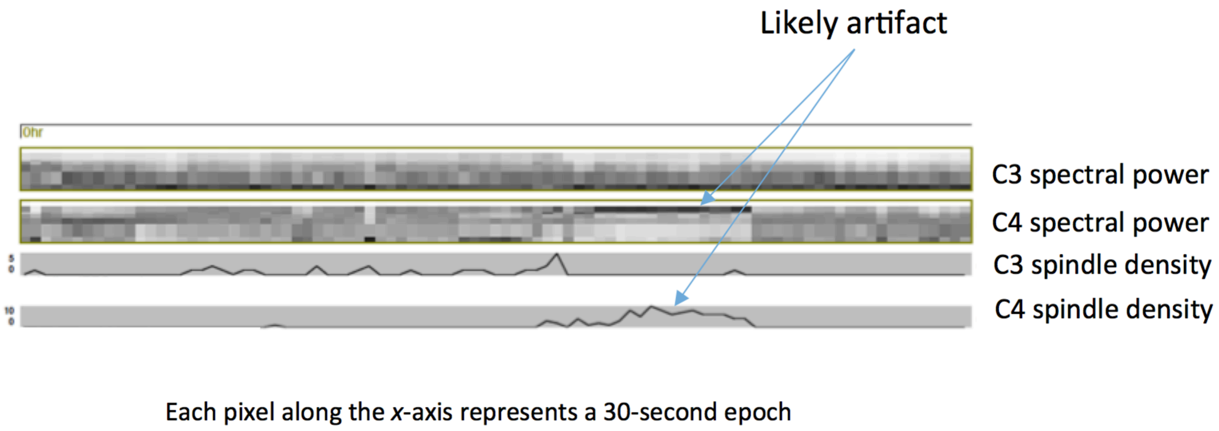


**Supplementary Figure 3. Illustration of temporally-clustered artifactual "spindles".** This Figure shows the post-filter N2 epochs for one example individual. For the two EEG channels, the top plots show a grayscale heatmap of per-epoch spectral power (darker is greater relative power). The bottom two plots show the per-epoch (30 seconds) number of spindles detected (the *y*-axis ranges from 0 to 5 for the first EEG channel, and from 0 to 10 for the second). As is evident, the second channel contained a likely artifact, for which a large number of putative spindles (almost ~20 per minute) were detected during a period that also has strong high-frequency activity (from the spectrogram). Even in such cases, the whole-night mean spindle density can still fall within a typical range, however, if no other spindles are detected. This instance, not already filtered out by the other automated filters, was flagged by considering the dispersion of the per-epoch spindle count.

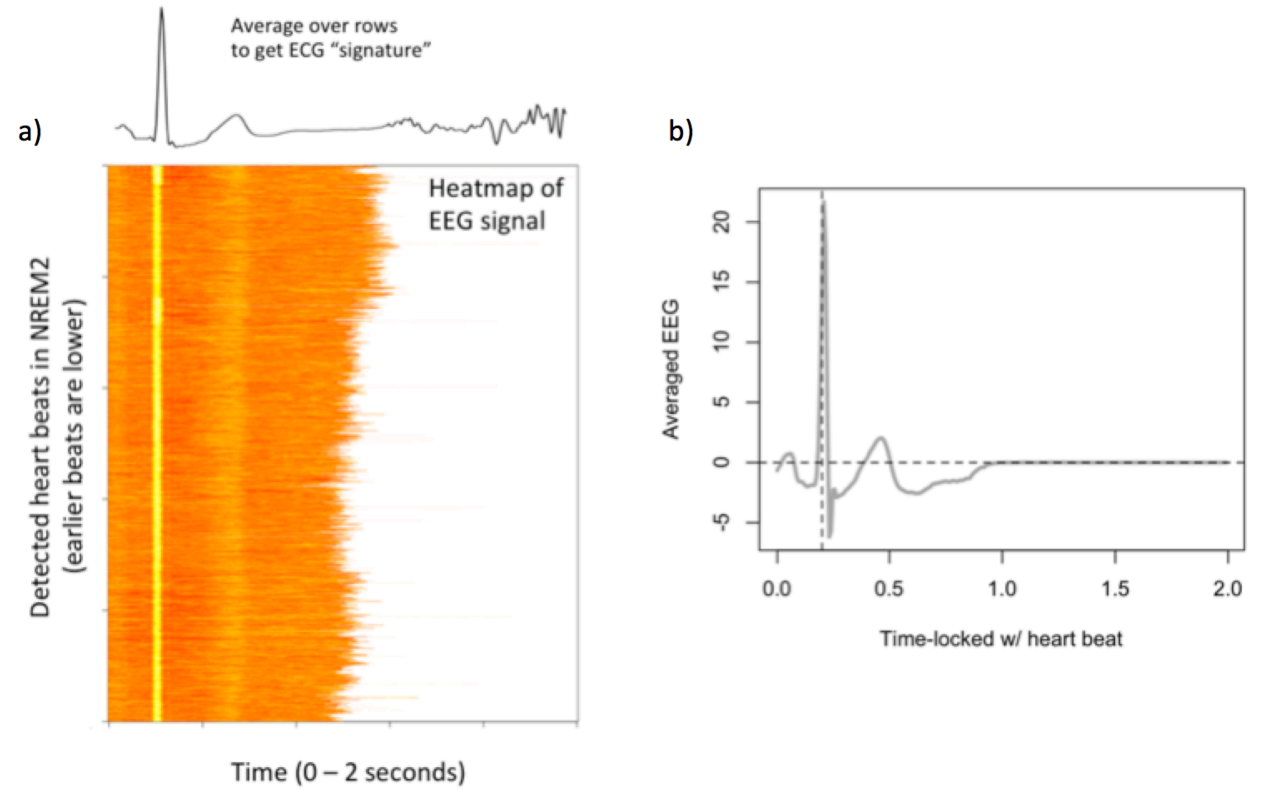


**Supplementary Figure 4. Illustration of individual signatures of cardiac (ECG) interference in the EEG.** a) For a given individual, we detected R peaks in the ECG and aligned all EEG intervals to be time-locked to the R peak, as shown in the heatmap. Averaging over these EEG intervals, we obtained an average signature to represent EEG activity that is temporarily locked to cardiac activity. b) Each time-point in the 2-second long signature is attenuated relative to the number of intervals that overlap it (i.e. the wiggle at the end of the signature was removed, as it represented only a small number of intervals). The scaled signature is subtracted from the EEG, time-locked to each R peak.

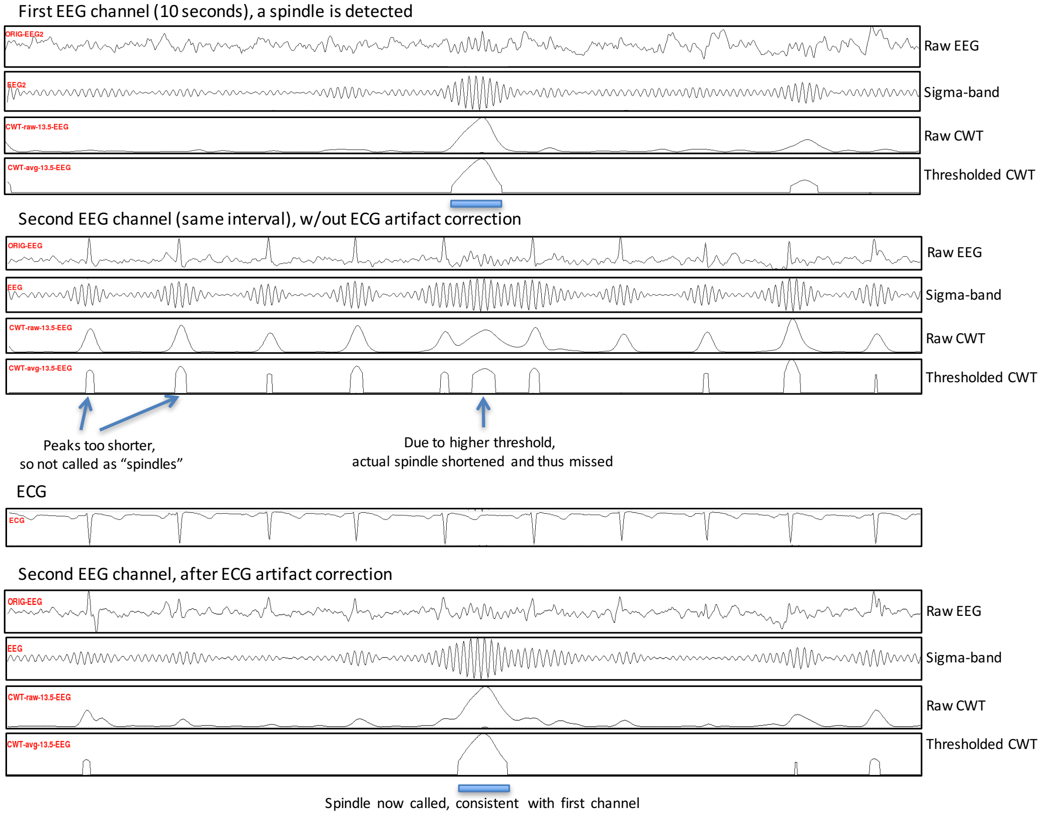


**Supplementary Figure 5. Illustration of ECG correction for one spindle/individual.** This Figure shows 10 seconds of EEG (two channels) and ECG for one individual. The top panel shows four signals: the raw EEG, the sigma band-pass filtered EEG, and the raw and thresholded coefficients from the wavelet analysis (i.e. from which spindles are detected). Here, for the first, uncontaminated EEG channel (C4), we see a boost in sigma power in the center of the plot, and a corresponding spindle detected (blue bar at the bottom of the panel). Below the top panel is an ECG channel, followed by similar plots for the second EEG channel (C3). For the second EEG channel, cardiac interference is clearly evident in the raw EEG, in this egregious example. The cardiac interference introduces many small pulses of sigma activity: although these are too short to be detected as spindles, they nonetheless raise the mean level of sigma activity (or the corresponding wavelet coefficient), leading to a reduced above-threshold interval over the putative spindle, meaning it is not detected on the second channel. The bottom panel shows the same plots for the second EEG channel, but after the ECG correction step described above. Although in this example not completely removed from the raw signal, the cardiac-induced sigma pulses are now greatly attenuated, leading to the successful detection of the (bilateral) spindle in this channel (C3), as was the case for the other (C4). For this particular individual, in whom we observed marked cardiac interference in one of the EEG channels, the correction had a profound effect on estimated spindle density. Prior to correction, the estimates were 0.05 and 1.97 for the two EEG channels. Post correction, the estimates were more concordant, at 2.12 and 1.97. To the extent that an EEG channel does not show any coherence with the ECG, this correction will have no effect, as illustrated by the unchanged value (1.97 spindles per minute) for other, artifact-free channel.

a)

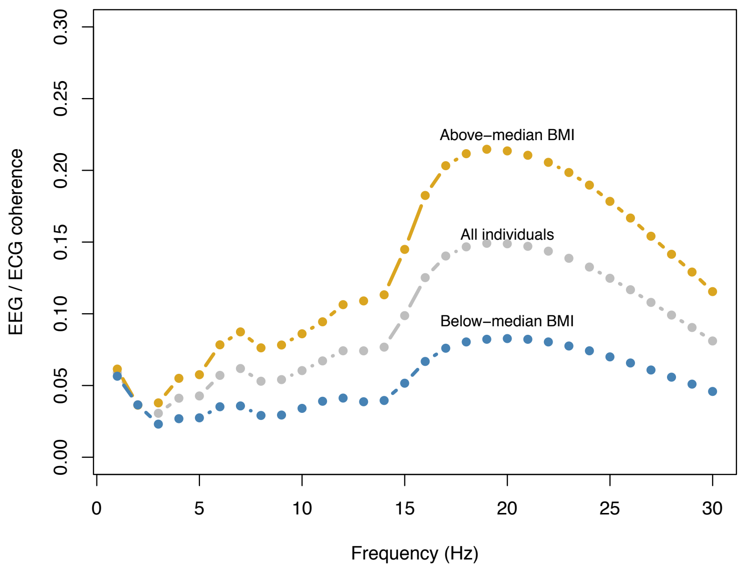


b)

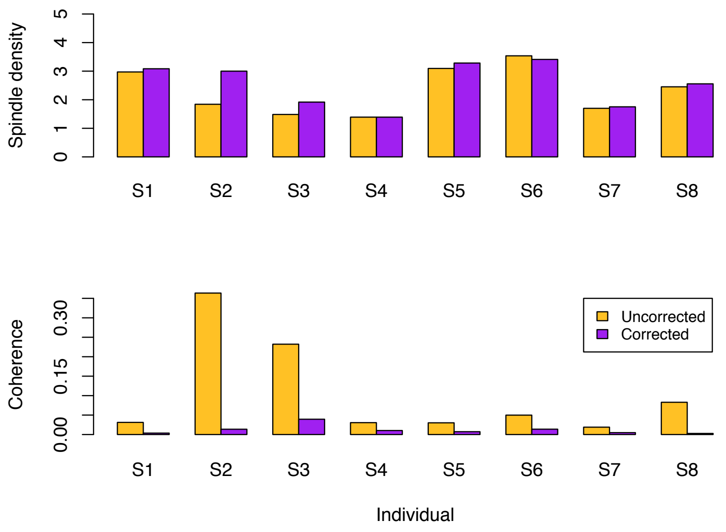


**Supplementary Figure 6. Impact of cardiac signal contamination and body mass index on detection of spindles in the EEG.** a) EEG/ECG spectral coherence at different frequencies (*x*-axis) stratified by high and low body mass index. b) Estimates of spindle density pre- and post-correction for potential cardiac interference in the EEG, and sigma-band EEG/ECG coherence, from the eight subjects in the DREAMS database. Individuals with higher sigma-band coherence pre-correction show substantive increases in their revised spindle rates (S2 and S3), consistent with the effect of correction in the NSRR data.

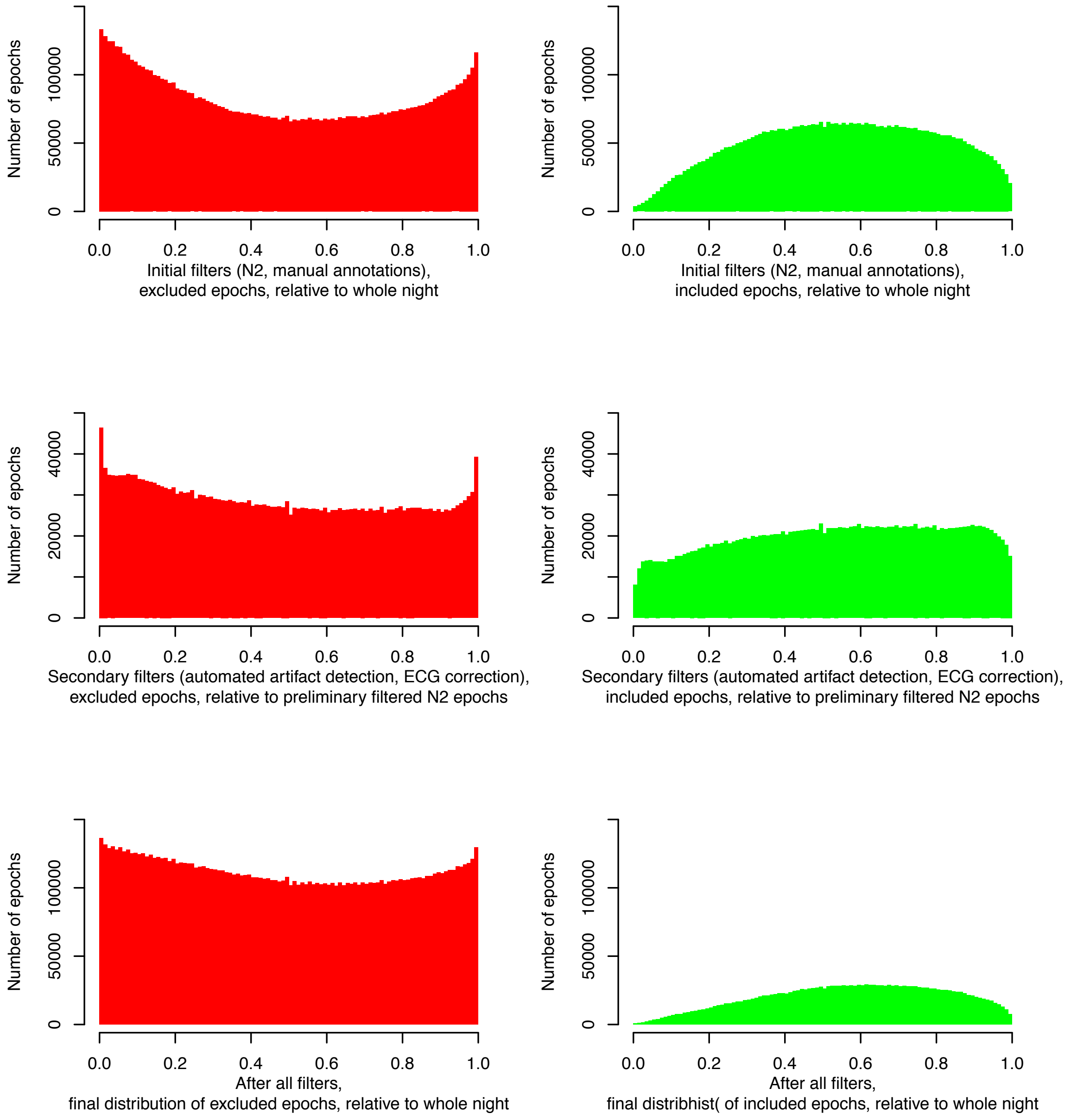


**Supplementary Figure 7. Distribution of included and excluded epochs across the night.** Red histograms represent the excluded epochs, green the included epochs. For the top row, the *x*-axis indicates the position of the epoch relative to the whole night; inclusion/exclusion is based on an epoch being scored as N2, and not having a manually noted annotation for a movement, arousal or signal artifact. Starting with the remaining N2 epochs (i.e. as depicted in the top right plot), the second row shows the temporal distribution of epochs excluded by the subsequent automated filtering. This was largely uniform across the span of N2, with the exception of spikes very near the beginning and end. Finally, the bottom row shows the overall distribution of excluded/included epochs from all rounds of filtering, relative to the whole night.

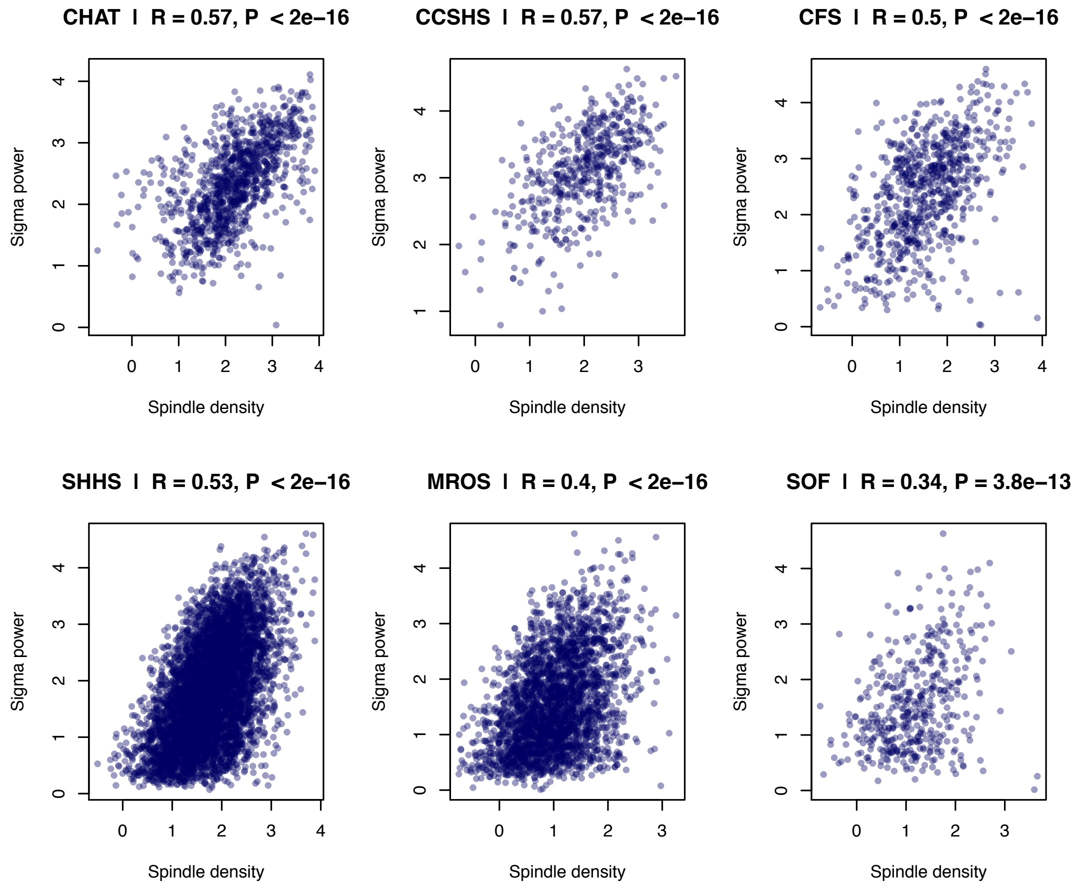


**Supplementary Figure 8. Spindle density and sigma power, stratified by study.** Stratified by study, the relationship between average spindle density and sigma power (absolute power, log-transformed) across all N2 sleep.

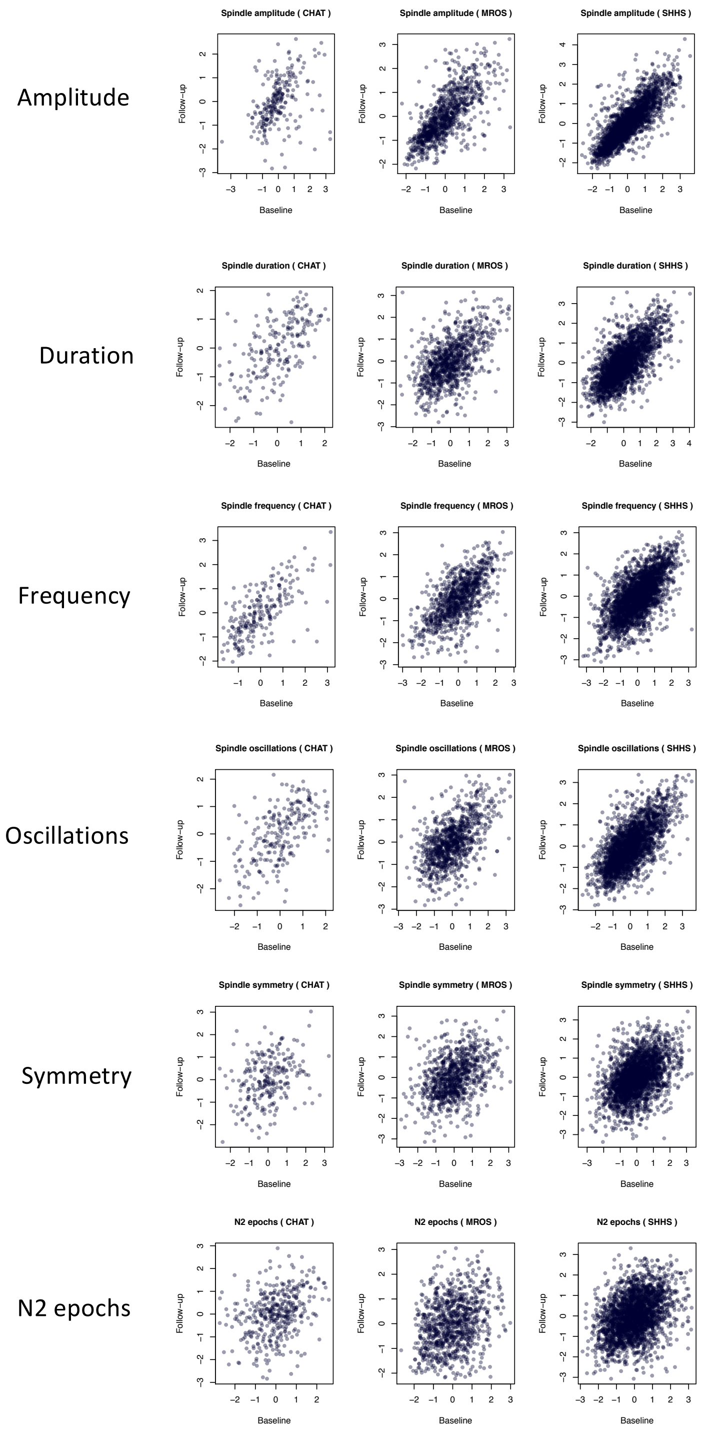


**Supplementary Figure 9. Test/retest scatter plots for spindle properties.** Scatter plots showing baseline/follow-up bivariate distributions for various spindle properties, and also the number of N2 epochs retained for analysis, for the three studies with repeated PSGs (CHAT, SHHS and MrOS). Correlations for each measure are given in **Supplementary Table 4**. Test/retest plots for spindle density are given in **Figure 1**.

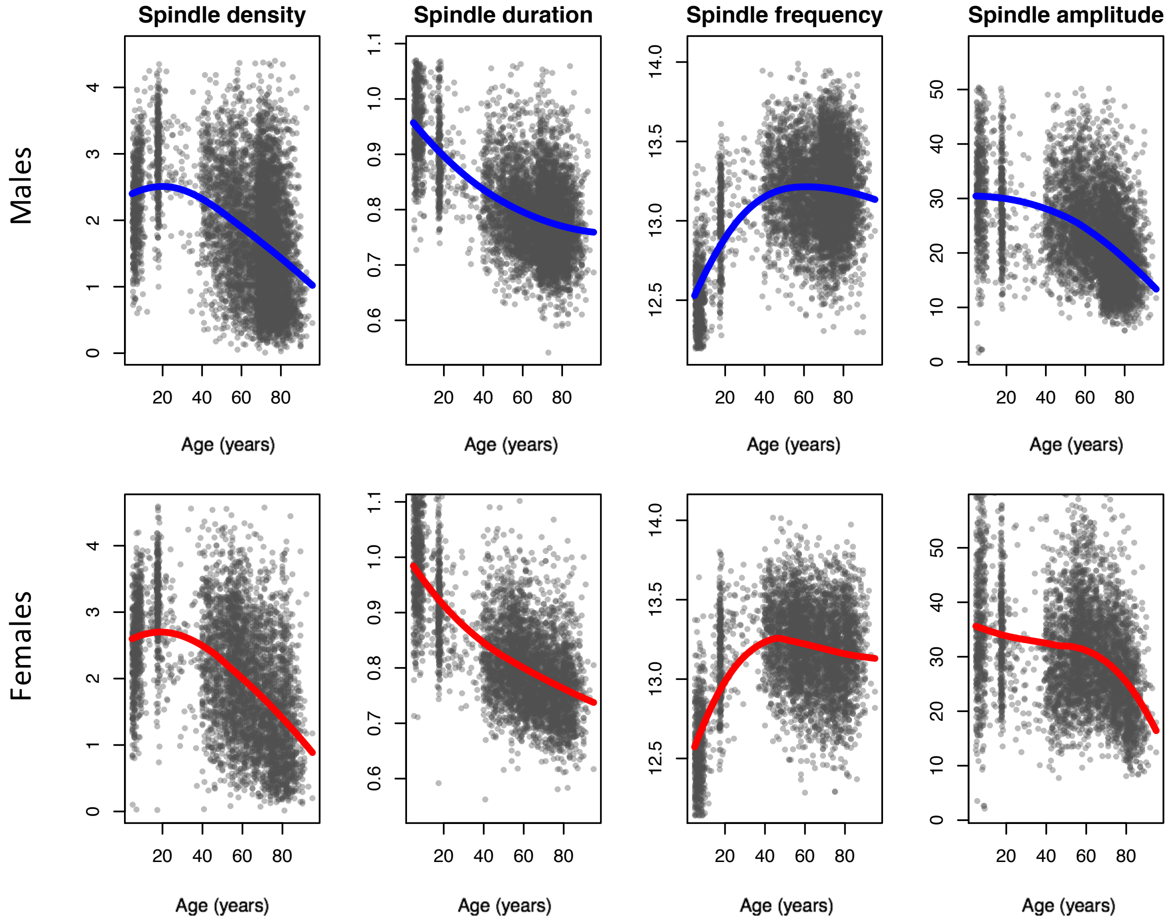


**Supplementary Figure 10. Life course trajectories for spindle properties, in males and females.** Measures are plotted against age in years (*x*-axis) with a smooth curve fitted by loess. Life course trajectories of spindle properties were also statistically analyzed cross-sectionally within study (**Supplementary Table 8**) and longitudinally, for individuals with repeated measures (**Supplementary Table 9**).

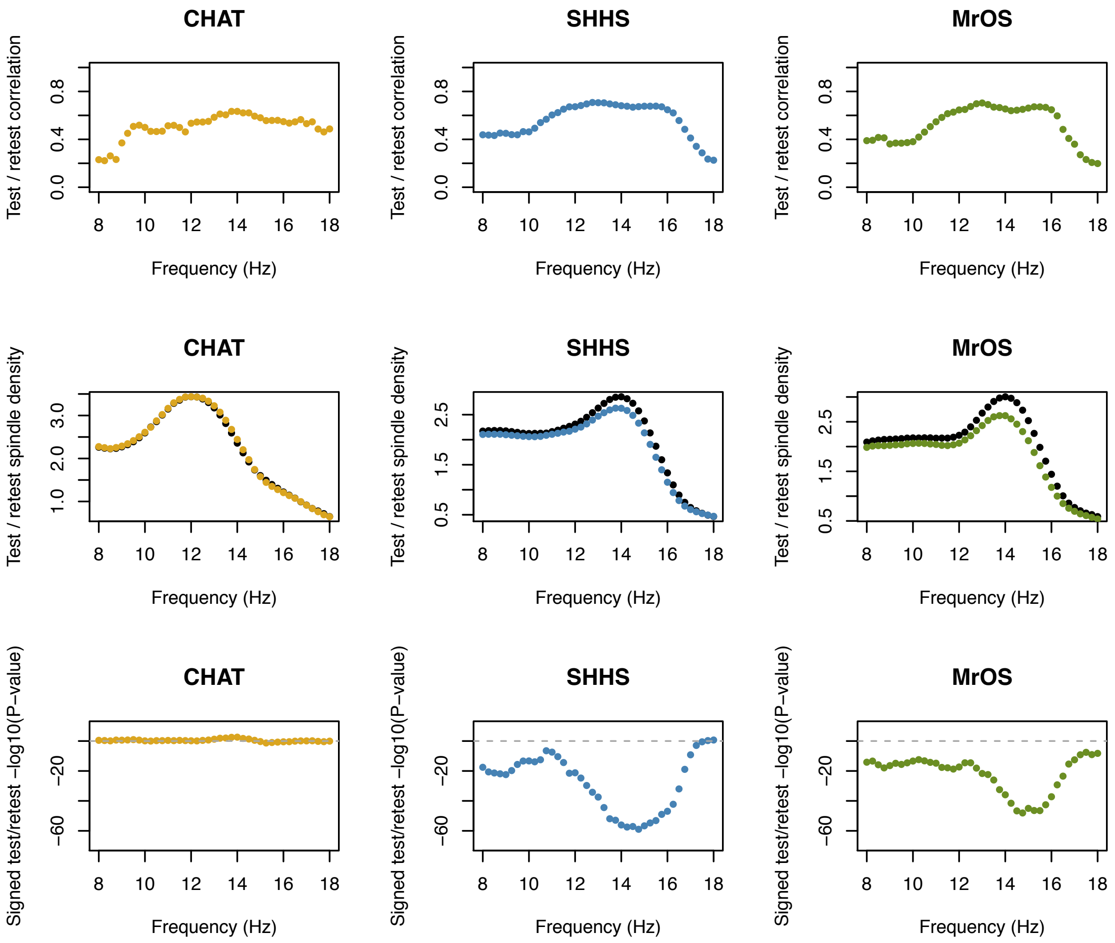


**Supplementary Figure 11. Test/retest correlations, means and tests for statistical differences for spindle density, stratified by study and spindle frequency.** For the three studies with repeated PSGs, the top rows show test/retest correlations (all highly significant) for estimates of spindle density across a range of targeted frequencies (8 to 18 Hz). The middle row of plots shows the baseline (black dots) versus follow-up (colored dots) mean spindle densities. The bottom row shows the significance (–$\log_{10}(p)$, negative values denote decreases in density at retest) for the difference in densities from baseline to follow-up. Note that for CHAT (which had a relatively brief retest interval of only ~6 months), there was in fact a modest within-individual increase in spindle density for spindles detected with $F_C$ values around 13-14 Hz, although this is not visible in the Figure, as the scale of the $y$-axis is fixed across all three studies.

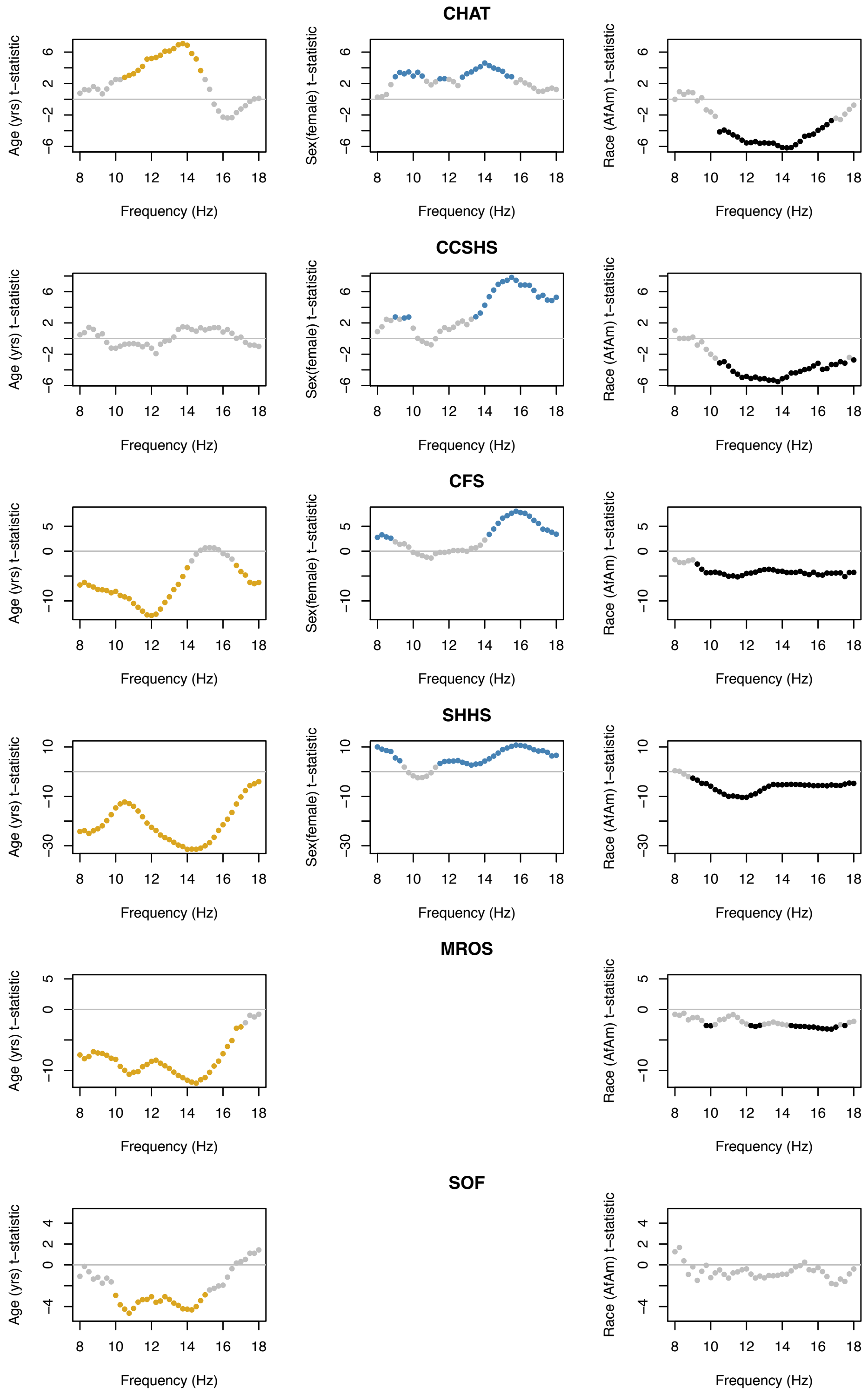


**Supplementary Figure 12. Demographic predictors of spindle density across a range of frequencies, stratified by study.** This Figure shows *t*-statistics for age, sex and race (left, middle and right columns) stratified by the six studies (rows). Results were based on a regression of spindle density (for a given $F_C$) on age, sex, race, arousal index and AHI. Colored points indicate that result was significant at $p < 0.01$.

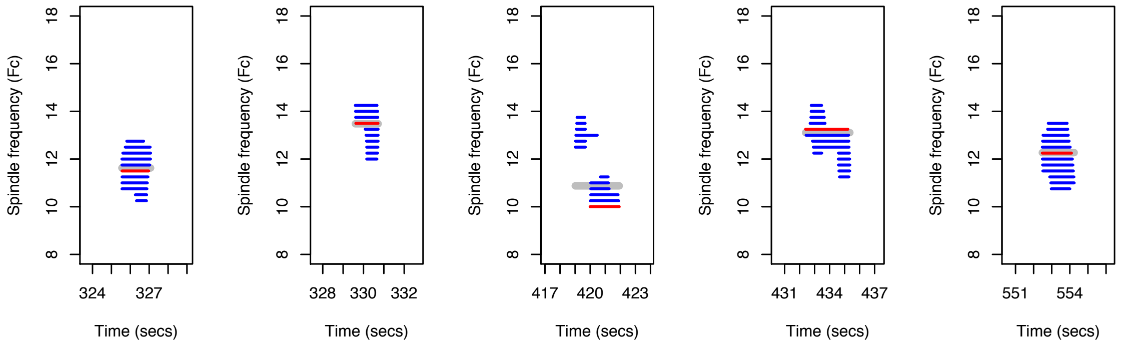


**Supplementary Figure 13. Examples of merging overlapping spindles across different $F_C$.** Five examples of a single unique, discrete spindle event being detected based on overlapping spindles from a frequency-dependent analysis (here 10 to 16 Hz in 1 Hz intervals). The *y*-axis represents the targeted frequency ($F_C$); blue lines indicate that a spindle was detected at that position by the corresponding analysis. The red line indicates the $F_C$ that yielded the maximum evidence for a spindle (based on the wavelet coefficients). The gray line represents the average spindle frequency, calculated as a weighted mean across the overlapping pool of spindles. Any two detected spindles were defined as representing the same true spindle if either a) their intersection was more than 50% of their union, or b) more than 80% of any one spindle was overlapped by the other.

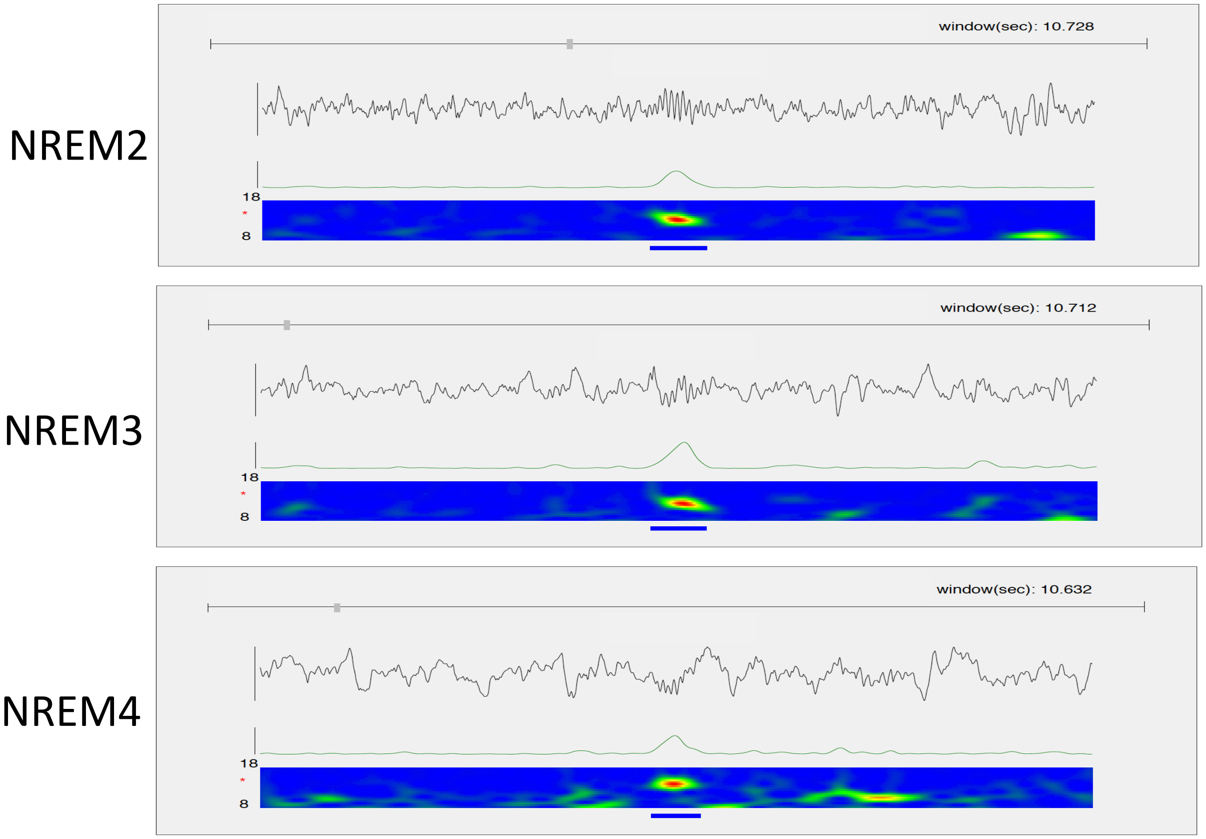


**Supplementary Figure 14. NREM2, NREM3 & NREM4 spindles.** This Figure shows three example spindles in the same individual from the older Rechtschaffen and Kales staging: NREM 2, NREM3 and NREM4 (only used in the SHHS). To consider spindles in NREM3 and NREM4 more generally, we selected up to 10 minutes of NREM3 (or NREM4) sleep per individual, only taking epochs that were both preceded and followed by two other NREM3 (or NREM4) epochs. We estimated spindle density during NREM2, NREM3 and NREM4 for the 315 SHHS individuals with at least 5 minutes (10 epochs) of each stage, using the same analytic procedure as in the original N2 analysis. In this subset, spindles were present in all stages, but significantly ($p < 10^{-6}$) lower for NREM4 (mean density 1.03, SD 0.77) versus NREM3 (mean 1.57, SD 0.96) versus NREM2 (mean 1.78, SD 0.98) sleep. Importantly, spindle density for NREM4 was highly correlated with NREM3 ($r$=0.79) and NREM2 ($r$=0.64) estimates. The primary demographic associations for NREM4 spindles were similar: males had 0.3 fewer spindles per minute ($p$=0.04); every additional year of age was associated with 0.02 fewer spindles per minute ($p = 4\times10^{-5}$). NREM 4 spindles were also significantly shorter and had lower amplitude than NREM3 spindles, which were in turn shorter and of lower amplitude than NREM2 spindles ($p < 10^{-6}$). As with density, we observed significant correlations in individual's NREM2, NREM3 and NREM4 mean spindle duration and amplitude. Taken together, these results suggest that the spindles automatically detected during slow-wave sleep, although unlikely to be flagged by a standard visual inspection of the raw EEG, likely reflect true, biological spindle activity.

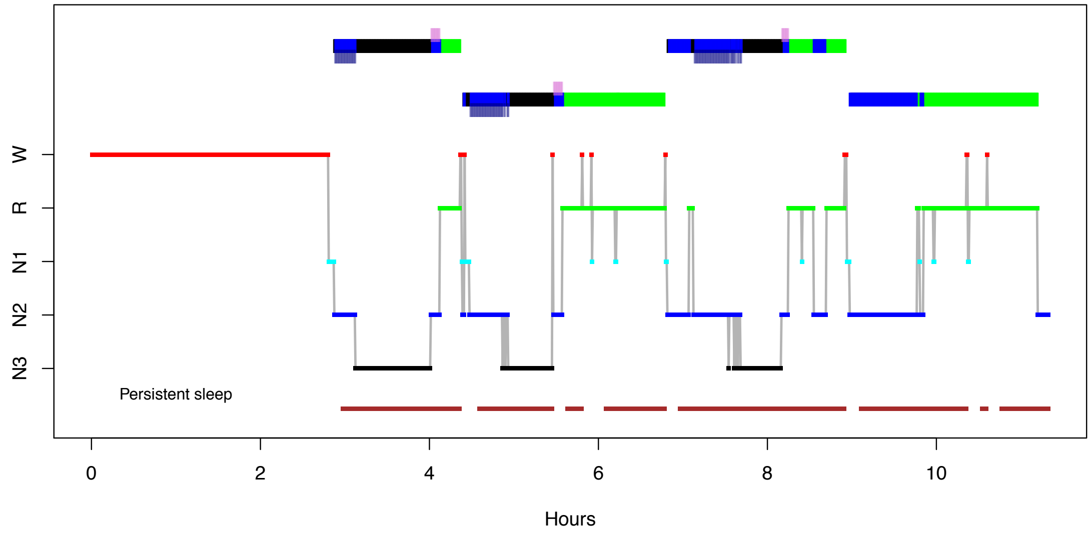


**Supplementary Figure 15. Example hypnogram with sleep cycles and ascending/descending N2 epochs annotated.** An example hypnogram, annotated with sleep cycles (four bars above the hypnogram). Blue, black and green represent stages N2, N3 and R respectively. The tags above and below the cycle bars indicate that the N2 epoch is defined as ascending (transitioning from N3 to N1/R/W) or descending (from N1/R/W to N3). The bottom brown line indicates persistent sleep.

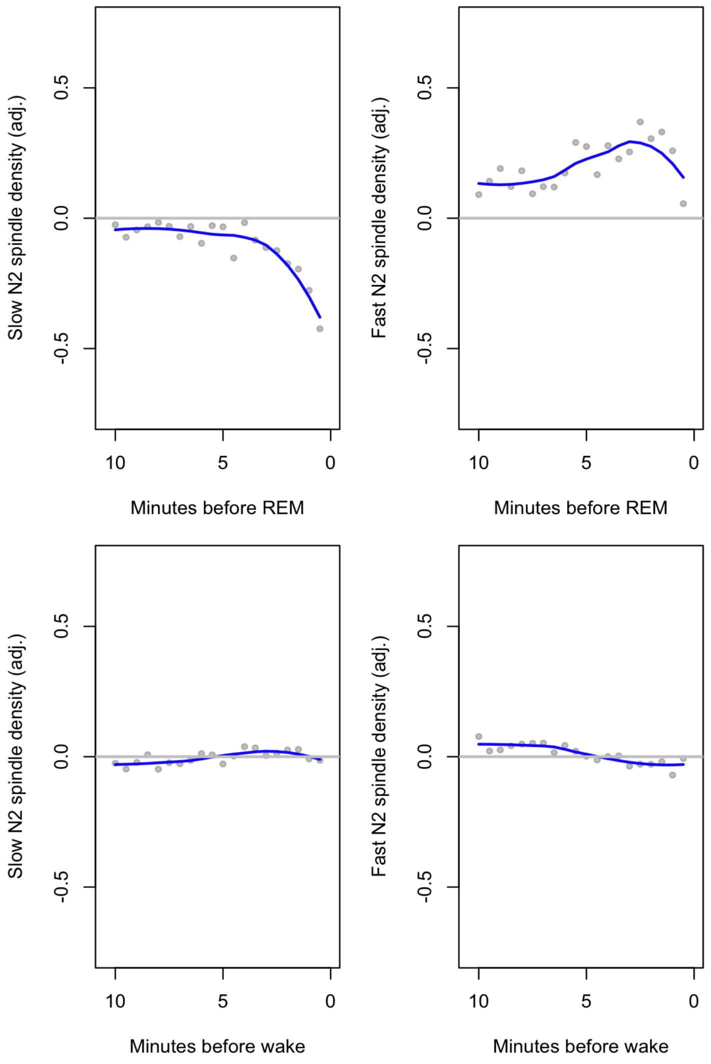


**Supplementary Figure 16. Sleep spindles near NREM/REM and NREM/wake transitions.** This Figure shows the mean per-epoch N2 spindle density (spindles per minute, i.e. twice the number of observed spindles per 30-second epoch) for either slow or fast spindles ($F_C$ = 11 Hz or 15 Hz), as a function of the minutes of uninterrupted N2 sleep prior to a transition to either REM sleep or to wake (censored at 10 minutes). N2 epochs that transitioned to a state other than REM before transitioning to REM were not included in this analysis (likewise for the N2-wake transition). In other words, counting backwards from each new REM (or wake) epoch, all preceding N2 epochs were included until a non-N2 epoch was encountered. Spindle density was adjusted to account for the effects of sleep cycle number and position within the sleep cycle, as well as age, sex, study, race and individual, being based on the residuals from the linear mixed model with these terms as reported in **Supplementary Table 12**. Similar results were obtained if only N2 epochs during persistent sleep were analyzed. Likewise, results did not depend on whether the within-cycle covariate was the six-level factor, or entered as a continuous variable (either minutes or relative position within the cycle, scaled 0 to 1) with higher order terms (to the fifth). However, the reported effects for NREM/REM transitions in the top two plots were absent if we did not control for within-cycle effects.

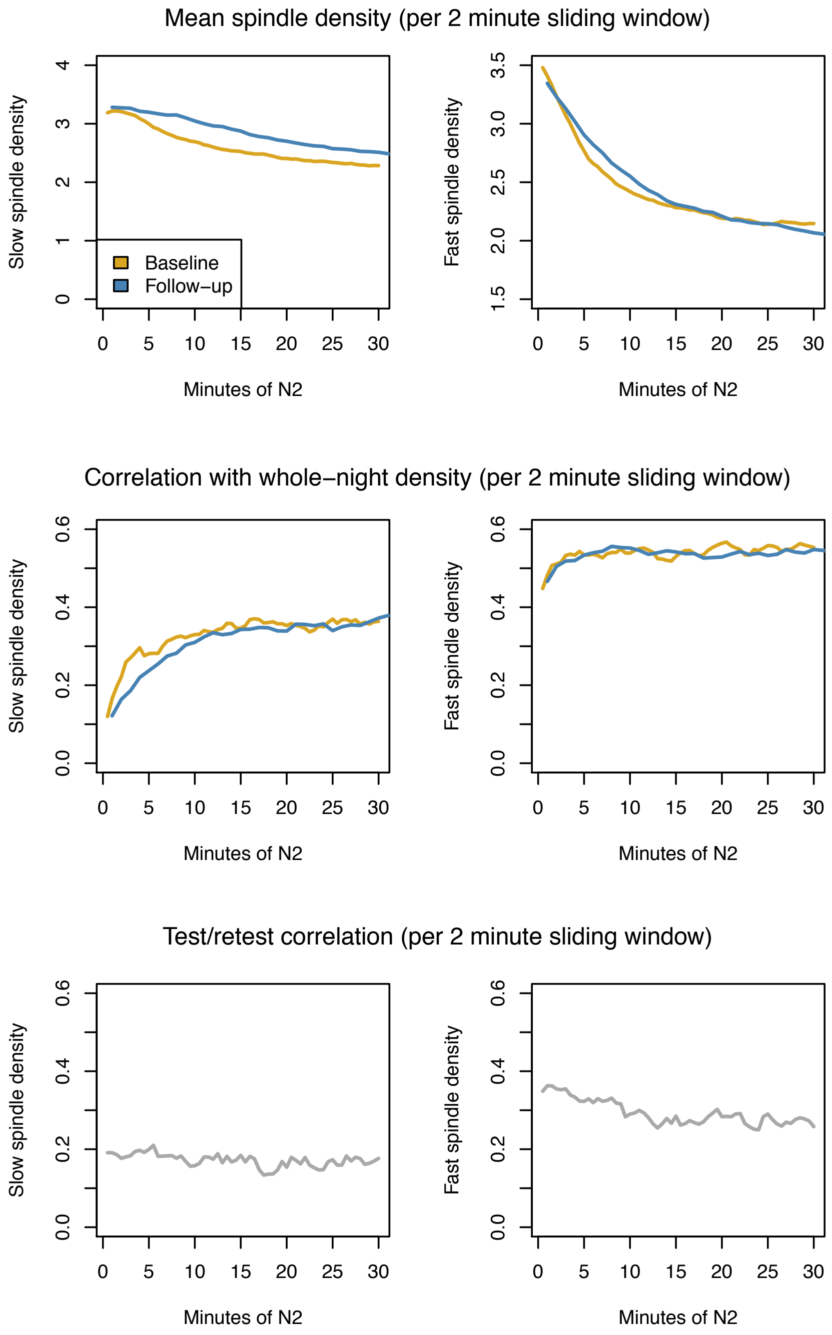


**Supplementary Figure 17. Sleep spindles during initial N2 sleep.** We considered slow ($F_C$ = 11 Hz, left column of plots) and fast spindles ($F_C$ = 15 Hz, right column) in a two-minute sliding window (i.e. mean spindle density from epoch *e* to epoch *e*+3) across the first 30 minutes of N2 sleep, based on the cumulative extent of N2 sleep in the primary analyses. We calculated a) mean spindle density (top row of plots), b) its correlation to whole-night N2 spindle density (middle row), and c) its test/retest correlation (bottom row). Note: the absolute values of the correlations in the middle and bottom row plots will reflect epoch window size and mean spindle density, leading to different degrees of measurement error; as such, the absolute values should not be interpreted directly.

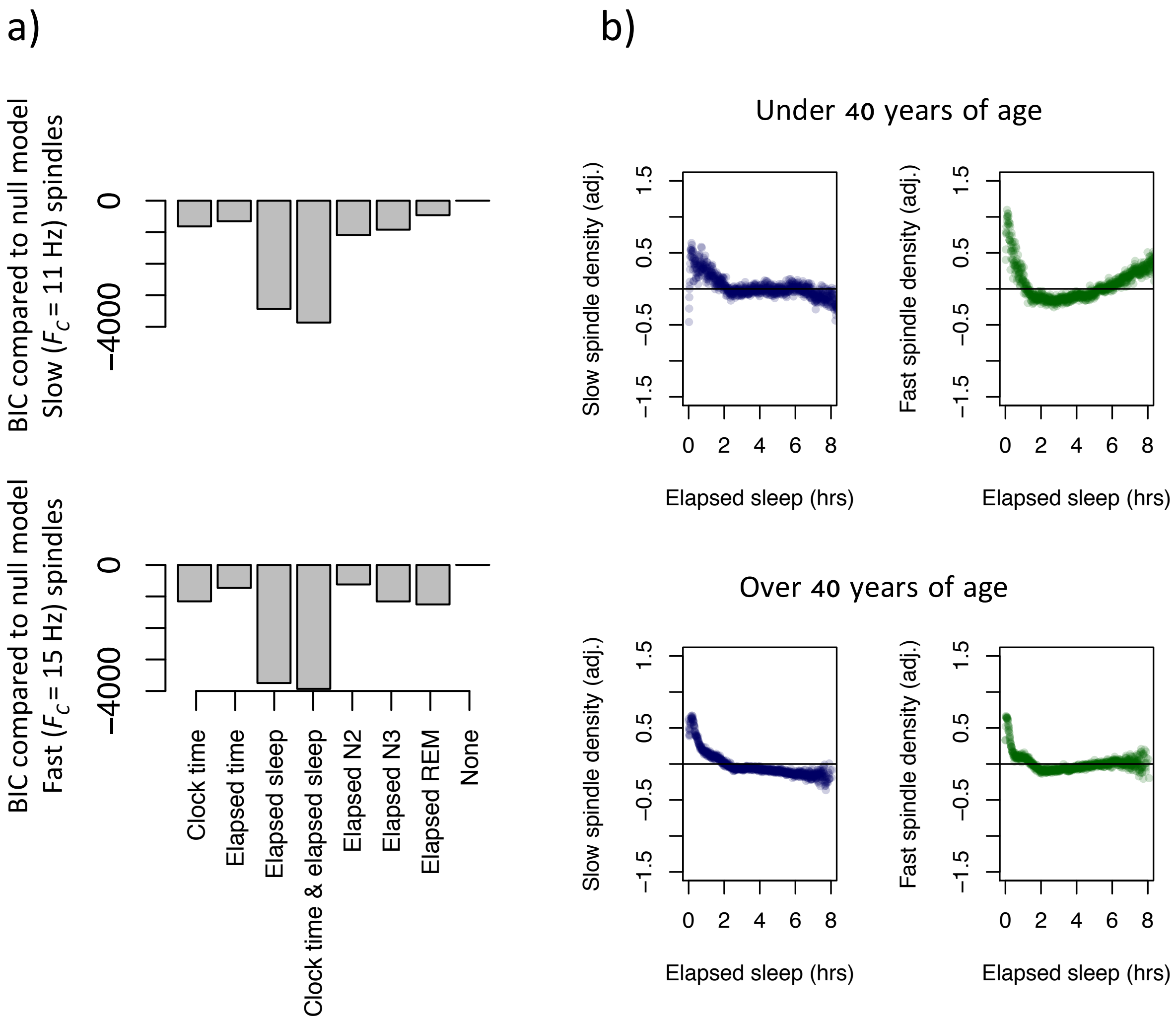


**Supplementary Figure 18. Modeling spindle density dynamics across the night.** a) Bayesian Information Criterion (BIC) values from a series of models (see Methods) predicting per-epoch spindle density as a function of local clock time, elapsed time, elapsed sleep, elapsed N2, elapsed N3, elapsed REM or none of the above. Lower BIC values imply a better model fit. For both fast and slow spindles, of the above models, the elapsed sleep model provided the best fit, although this was further improved by adding local clock time. b) Per-epoch spindle density (spindles per minute, but mean-centered for each individual) plotted against elapsed sleep (in hours). The facets of spindle dynamics evident in the sleep cycle analysis (**Figure 3**) can also be seen here: i) a general peak of spindle activity within the early stages of N2 sleep, and following that, ii) a subsequent increase in fast spindles, stronger in younger individuals, and iii) a decline in slower spindles, stronger in older individuals.

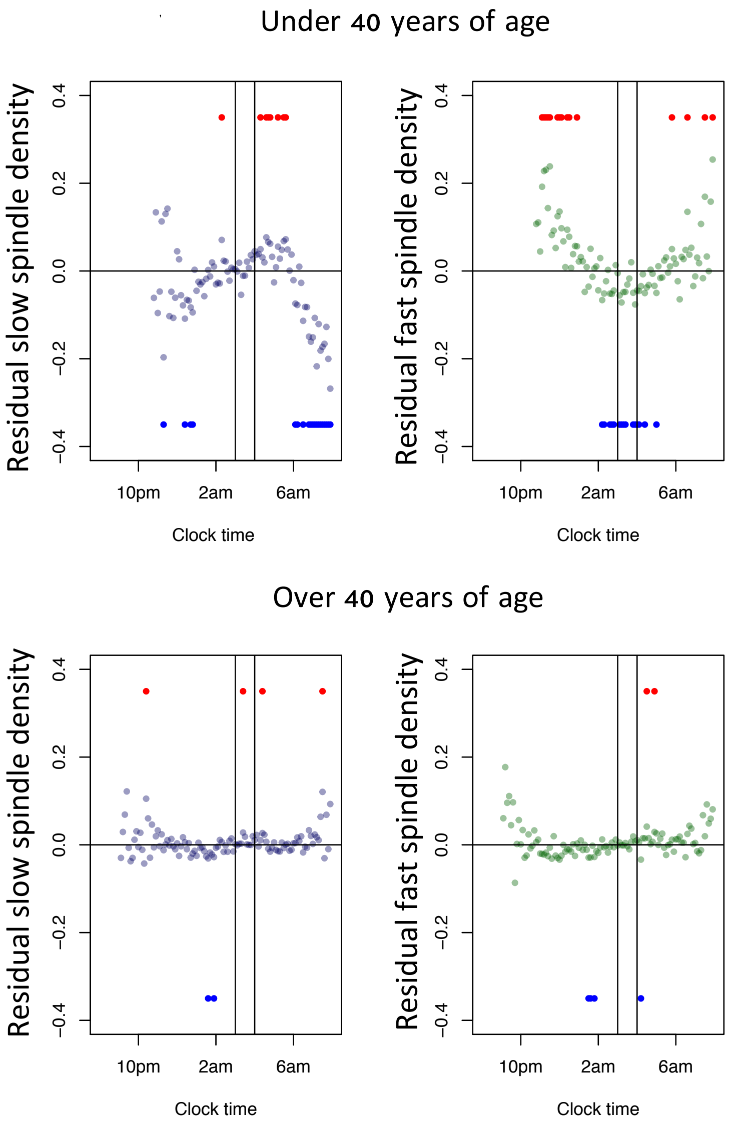


**Supplementary Figure 19. Circadian modulation of sleep spindles.** For fast and slow spindles separately, we extracted per-epoch spindle density residuals after accounting for the extent of elapsed sleep, as well as covariates for other ultradian and individual-level factors, namely sleep cycle number, within-cycle position, ascending/descending status, age, sex, study and race (models fit to all individuals, similar to those presented in **Supplementary Figure 18**). Here we conservatively included only epochs during persistent N2 sleep; furthermore, all epoch-level predictors allowed for an interaction with age, to account for age-dependent differences in spindle dynamics (results were robust to these decisions). By definition, these residuals were uncorrelated with elapsed sleep; they are plotted here against local clock time, separately for younger (under 40 years, mean ~12) and older (over 40 years, mean ~67) individuals. Only epochs between 8pm and 8am were included in analysis; epochs were pooled into 121 evenly-spaced bins for plotting; only bins with at least 200 observations were included in the plot, to reduce noise in estimates at earlier and later time-points that have few observations. Across all individuals/time-points, the residuals have a mean of 0; for each bin we performed a two-sided $t$-test to detect bins with residual spindle densities greater or less than expected. Red/blue points at the top/bottom of each plot indicate tests with $p$<0.01 for means greater/less than 0. The two vertical black lines in each plot indicate the interval from 3am to 4am, typically near the point of maximum melatonin levels.

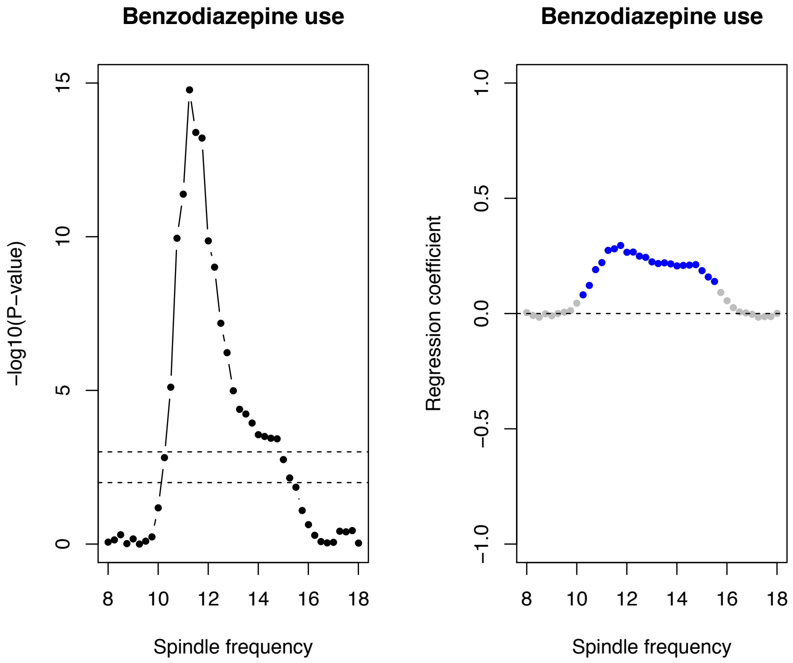


**Supplementary Figure 20. Association of benzodiazepine use and spindle density in SHHS.** From the frequency-dependent analysis, the effect of benzodiazepines on spindle density for a range of targeted spindle frequencies (*x*-axis). The right panel shows regression coefficients (for 0/1-coded medication use; blue dots indicate $p < 0.05$). The left panel shows the corresponding log-transformed *p*-values (dotted lines showing $p = 0.05$ and 0.01 thresholds).

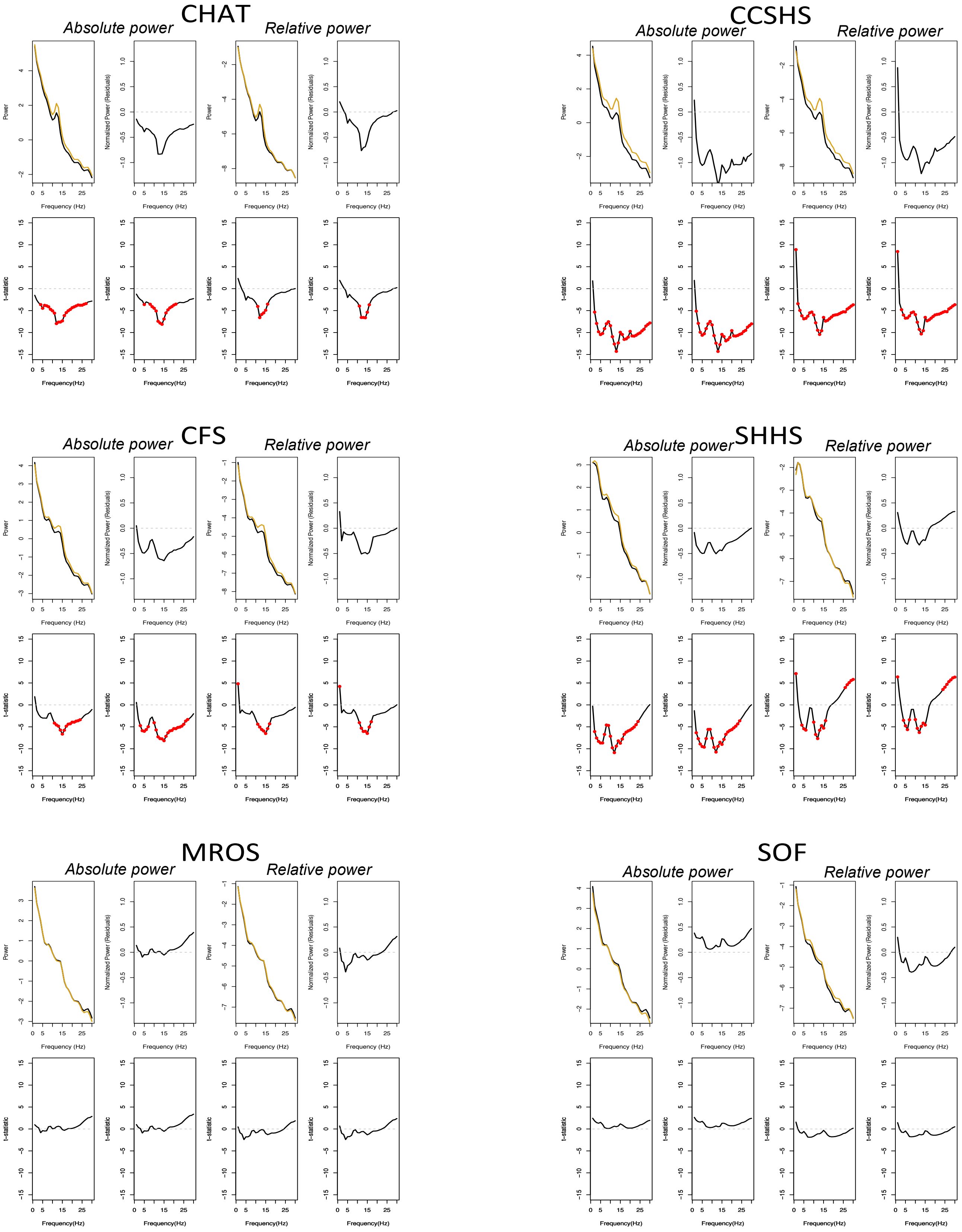


**Supplementary Figure 21** (see next page for legend)

**Supplementary Figure 21. Racial group differences in spectral power, stratified by study.** (Figure on previous page.) For each study, the eight plots indicate spectral power in blacks (black lines) and whites (tan lines). Each top row represents spectral power from 1 to 30 Hz (either absolute or relative, and either unadjusted or adjusted for common covariates (age, sex, BMI, arousal index and AHI). Each bottom row represents the *t*-statistic from an independent group *t*-test, red dots indicating a significant difference at $p < 0.001$; a negative *t*-statistic corresponds to a lower value in blacks compared to whites. For each study, the first column shows the absolute, unadjusted power spectrum; the second column shows the adjusted power spectrum, for which the outcome was the residual from a regression of spectral power on age (and higher order terms), sex, BMI, arousal index and AHI; results are normalized by the mean and standard deviation for whites at each frequency, so the black line shows the mean adjusted power for blacks relative to whites, in standard deviation units. The third and fourth columns show similar analyses but based on relative power (i.e. power values are normalized to sum to 1.0). Similar trends were observed for both absolute and relative power: namely that in younger studies (CHAT, CCSHS, CFS) blacks tended to show lower power especially in the sigma range; this effect was not found in the older studies (MrOS, SOF).

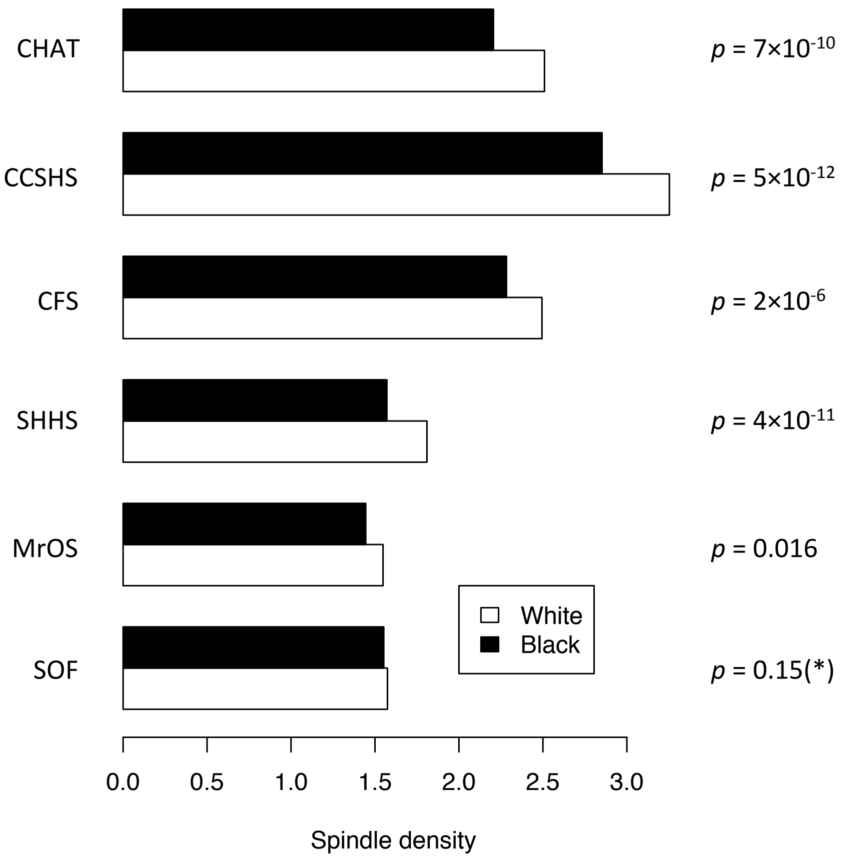


**Supplementary Figure 22. Racial group differences in spindle density, stratified by study.** The bar-plot shows mean spindle density (from the canonical $F_C$ = 13.5 Hz analysis) stratified by study and race (black, white). Consistent with the association between age, race and sigma power (**Supplementary Figure 21**, **Supplementary Table 19**), spindle density was on average lower in blacks than whites, although the effect was greatly attenuated in older individuals (**Supplementary Table 20**). The asterisk (*) denotes the very small number of non-white individuals (and thus statistical power for this contrast) in SOF.

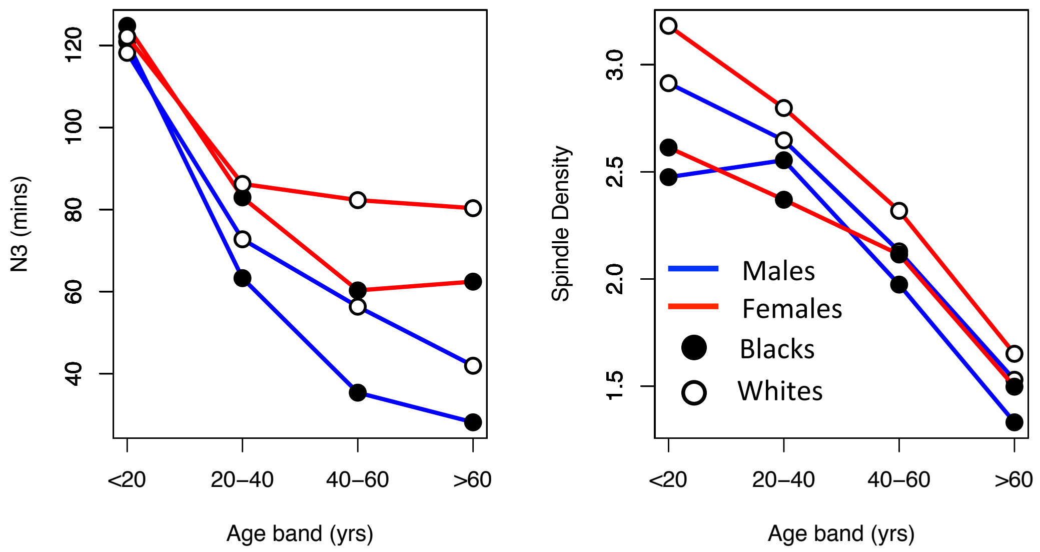


**Supplementary Figure 23. Duration of N3 sleep and spindle density as a function of age and race.** The left plot shows the duration of N3 sleep (minutes) as a function of age (grouped into four bands), sex (red/blue indicating female/male) and race (black/white). The right plot shows a similar breakdown for spindle density, from the canonical analysis ($F_C$= 13.5Hz).

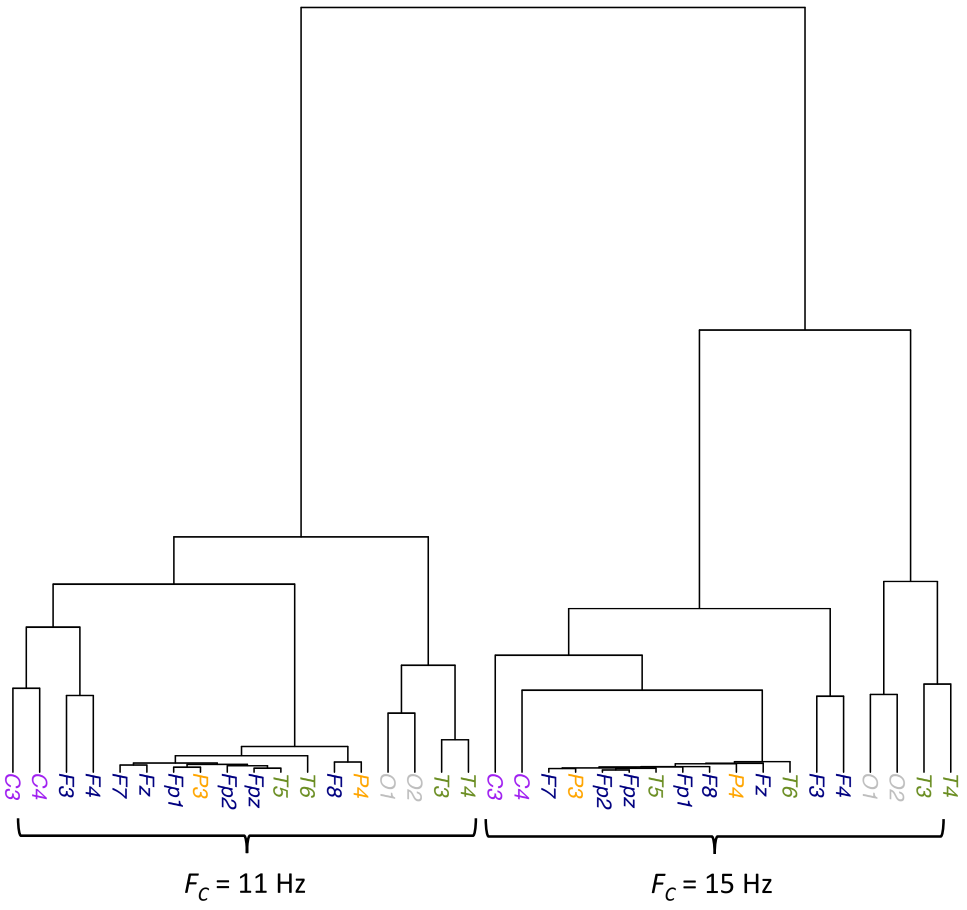


**Supplementary Figure 24. Dendrogram from a cluster analysis of individual spindle density estimates in the CHAT study.** In the subset of the CHAT study with data on 18 electrodes, we estimated mean spindle density (N2 sleep, separately for slow and fast spindles, $F_C$ = 11 and 15 Hz). Agglomerative clustering (average linkage method) was applied to a distance matrix defined as 1 – |*Rij*|, where *Rij* was the correlation in individual spindle density between electrode/frequency estimate *i* and *j*, where *i* and *j* index the 36 combinations of 18 electrodes and 2 target spindle frequencies (11 and 15 Hz). Electrode sites are color-coded by location (purple: central; gray: occipital; green: temporal; blue: frontal; orange: parietal). The corresponding dendrogram indicates that frequency, rather than topography, accounts for the principal axis of individual differences in within-channel spindle activity. Beyond that, contralateral electrodes tended to cluster together (i.e. C3 and C4, T3 and T4) as do most frontal electrodes. **Figure 6 and Supplementary Figure 25** gives a more focused presentation of the underlying correlation matrix, in terms of how well fast and slow spindles at C3/C4 predict spindle activity at other sites.

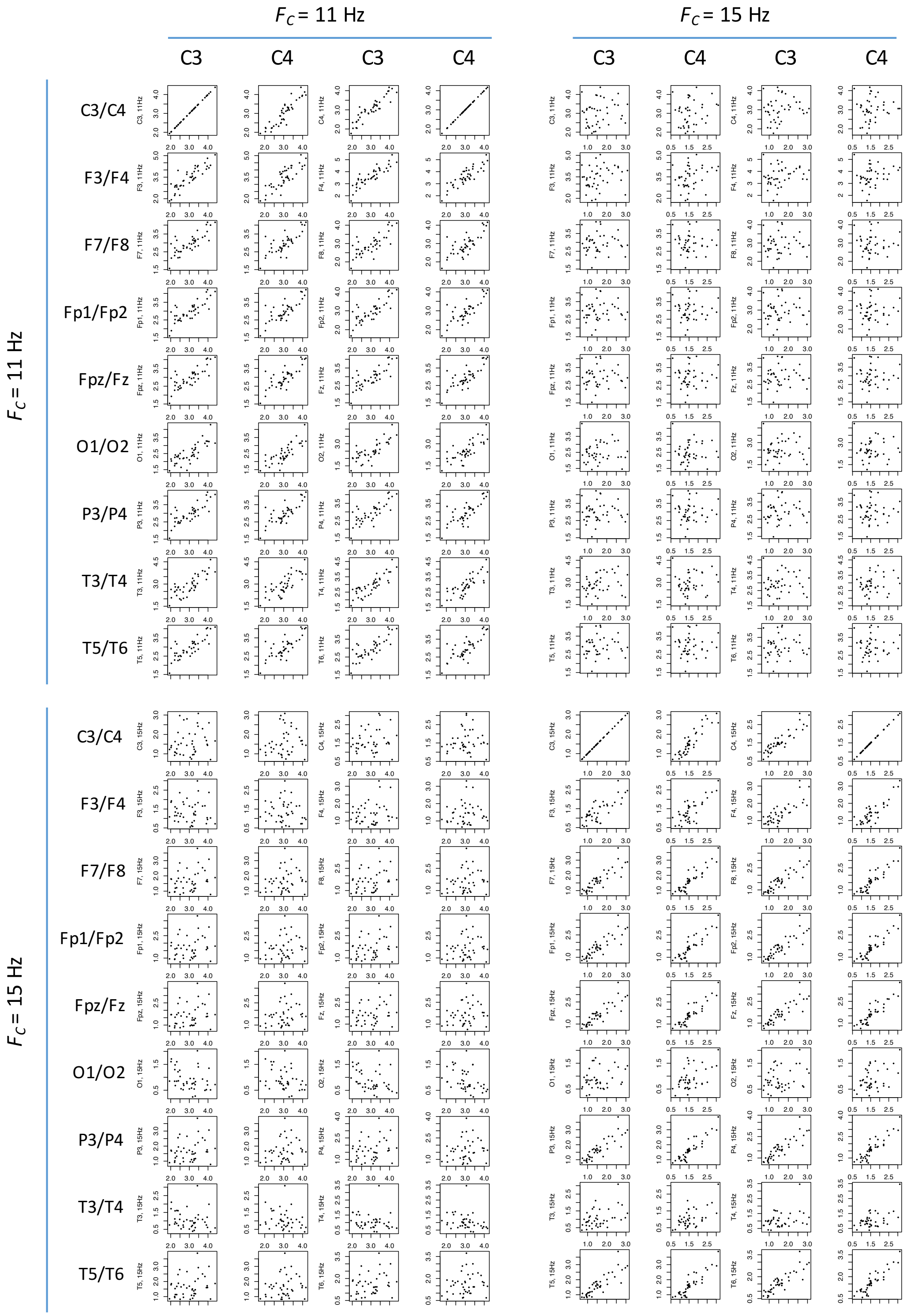


**Supplementary Figure 25. Scatter plots of N2 individual mean spindle density between channels for fast and slow spindles.** In a subset of the CHAT study, we calculated correlations (**Figure 6, Supplementary Figure 24**) in spindle density between C3/C4 and the 16 other electrodes, after removing outlier individuals and those with excessive leverage (see Methods). Each plot here corresponds to a point in **Figure 6c,d**.

a) $T_L$ low-amplitude spindles, $F_C$ = 13.5 Hz

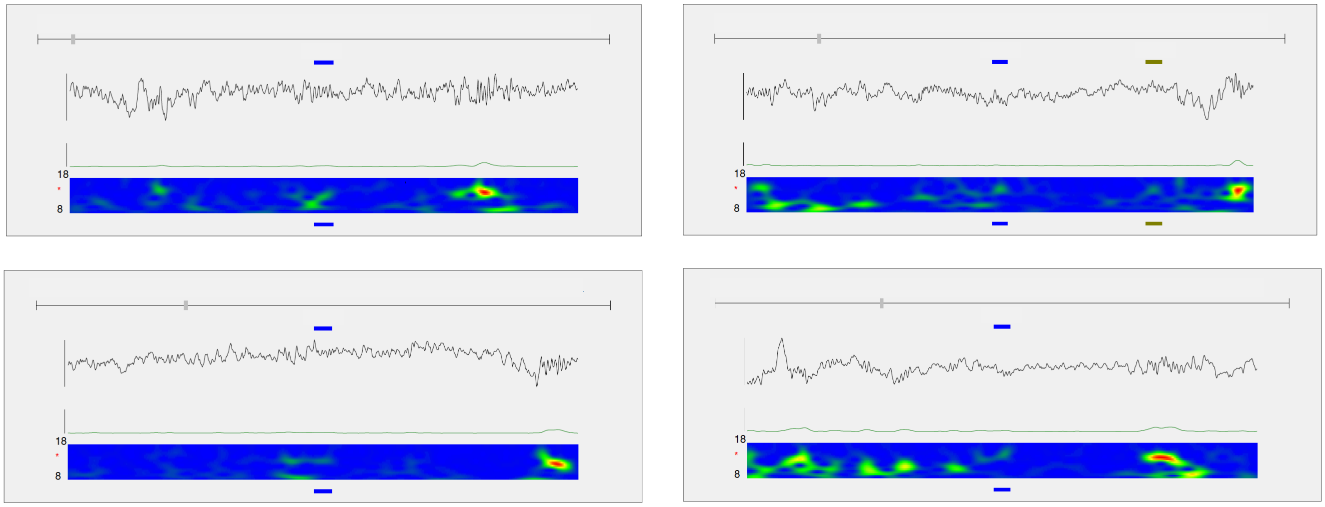


b) Default spindles, $F_C$ = 13.5 Hz

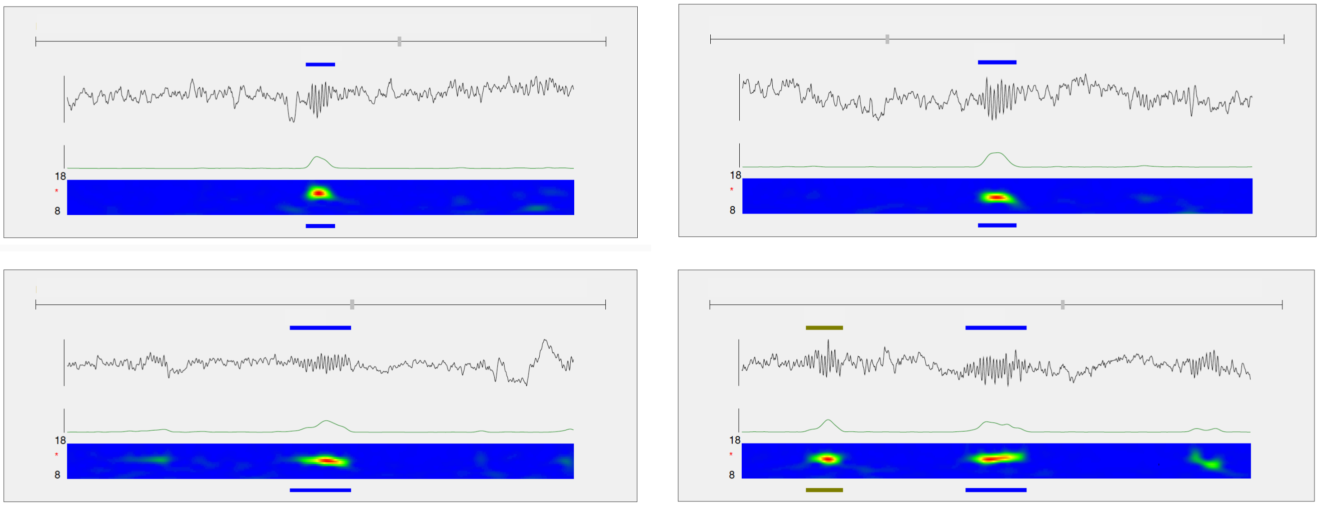


**Supplementary Figure 26. Examples of detected spindles from the low-amplitude ($T_L$) and default thresholds.** All spindles are from a single individual, targeting $F_C$ = 13.5 Hz. Each panel spans approximately 10 seconds. Although human raters would readily identify most spindles detected at default thresholds, low-amplitude spindles would likely be missed. Although this set inevitably has a lower signal-to-noise ratio, the non-random pattern of associations with relevant variables including age, race and benzodiazepine use suggest that these in fact represent the low end of the detectable spindle amplitude distribution (see **Supplementary Tables 30-32** and **Supplementary Figure 28**).

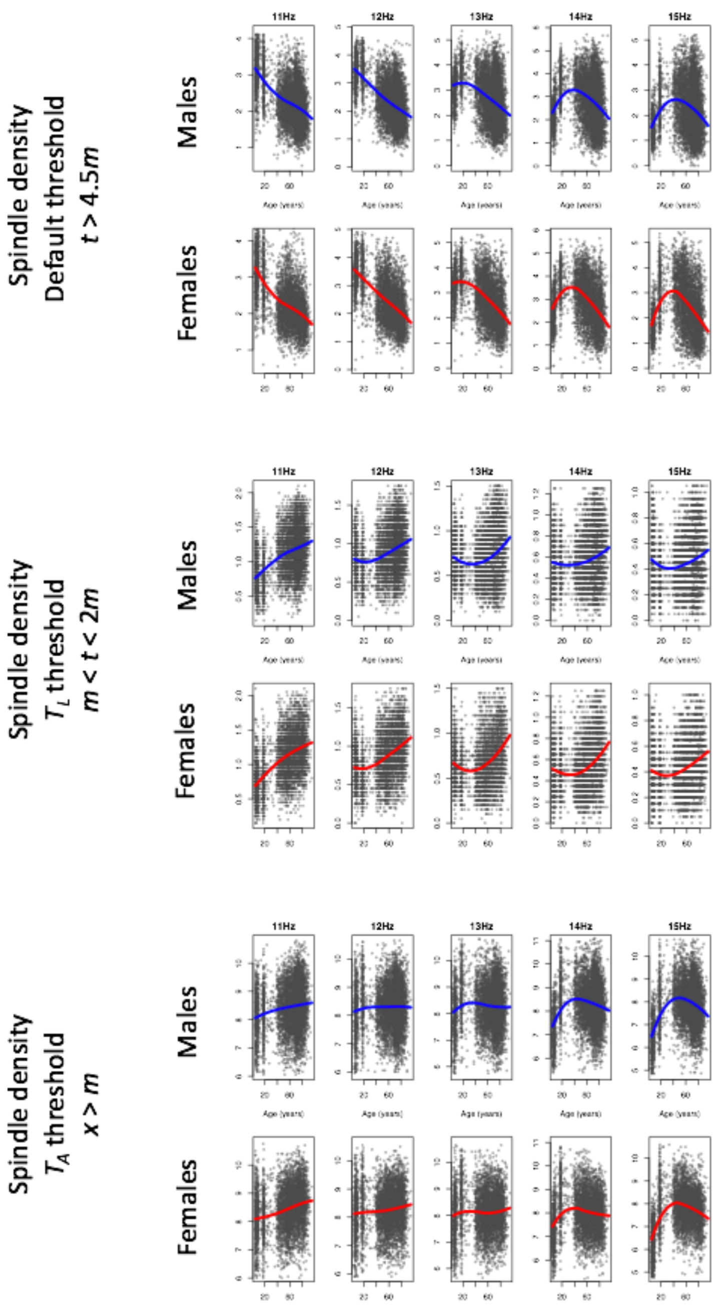


**Supplementary Figure 27. Varying spindle detection thresholds and life course trajectories for spindle density.** Each plot is similar to the plots of life course trajectories in **Supplementary Figure 10**, shown for males and females and a range of targeted frequencies. The top set of plots is based on the canonical analysis using the default amplitude criteria (4.5 times the mean). The middle set of plots corresponds to the $T_L$ analysis, in which spindles were detected only for signals between 1 and 2 times the mean, to detect only low-amplitude spindles. The lower set of plots corresponds to the broader $T_A$ threshold (spindles detected above the mean). Focusing on fast (15 Hz) spindles, whereas high amplitude spindles showed an inverted U-shaped association with age, low amplitude spindles showed an opposite, U-shaped association. This is consistent with a model in which spindle amplitude, rather than spindle density *per se*, is what primarily changes with increasing age (see **Supplementary Figure 28**).

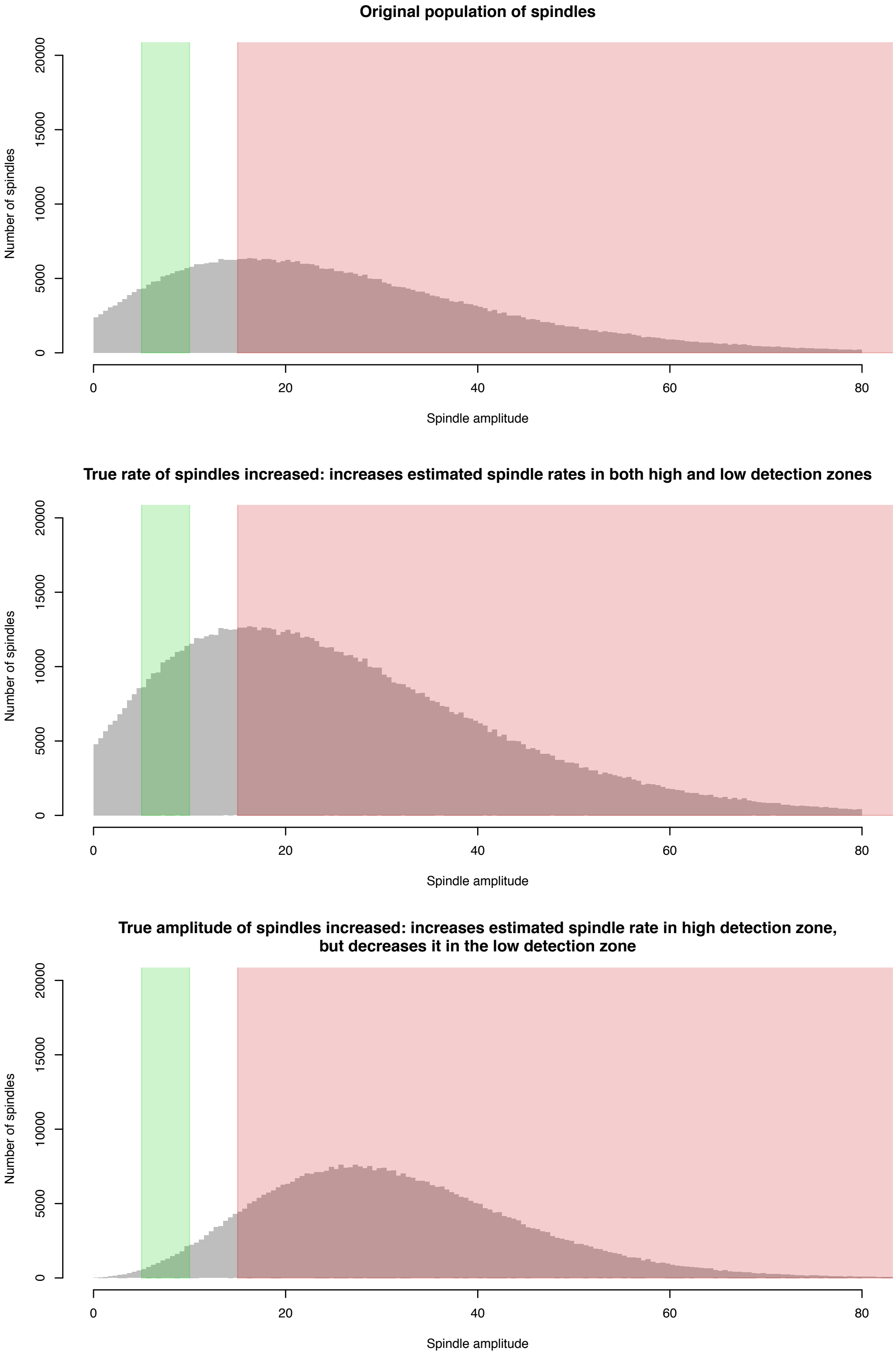


**Supplementary Figure 28.** (Legend on next page)

**Supplementary Figure 28. The relationship between spindle density, amplitude and detection threshold.** (Figure on previous page.) In this cartoon, we consider a hypothetical distribution of true spindle amplitudes as measured by scalp EEG (top panel, gray histogram), which we assume has some non-trivial degree of spread in terms of amplitude. The red zone represents the default high-amplitude detection zone for spindles (i.e. above 15 μV in this instance). The green zone represents a low-amplitude detection zone (i.e. between 5 and 10 μV in this example), which can be thought of as conceptually similar to the $T_L$ analyses reported above. (Note: our pipeline does not directly threshold spindles based on the signal amplitude, rather, it uses the relative magnitude of the wavelet coefficients; we use spindle amplitude here for simplicity of presentation.) We consider two possible changes in the true state of affairs for the population of spindles (e.g. if looking at a different group of individuals, for example). In the first instance, the rate of spindles increases but the average amplitude of the spindles does not change. Here we expect to see more spindles in both the low (green) and high (red) detection zones, and thus higher estimate of spindle density in both cases. An alternative scenario (bottom figure) posits no change in the true rate of spindles, but instead spindles tend to be stronger, meaning that the amplitude distribution is shifted to the right. This leads to qualitatively different predictions for the estimates of spindle density at the two detection zones: we'd expect to see a greater density of high-amplitude spindles, but a lower density of low amplitude spindles, even though the true spindle density is unchanged. Naturally, in reality the true distribution of spindle amplitude will be unknown and so different detection thresholds may show different patterns of changes in estimated spindle density as a result of varying spindle amplitude.

| | CHAT | CCSHS | CFS | SHHS | MrOS | SOF |
|---|---|---|---|---|---|---|
| Mean sigma EEG/ECG coherence ($C_S$) | 0.018 | 0.032 | 0.046 | 0.128 | 0.027 | 0.022 |
| Mean BMI | 18.8 | 24.9 | 30.4 | 28.0 | 27.2 | 27.7 |
| Correlation between $C_S$ and BMI | 0.065 | 0.372 | 0.410 | 0.389 | 0.311 | 0.047 |
| *P* value | 0.04 | $< 10^{-15}$ | $< 10^{-15}$ | $< 10^{-15}$ | $< 10^{-15}$ | 0.33 |
| Mean HR | 77.26 | 60.50 | 66.19 | 63.14 | 59.33 | 62.62 |
| Correlation between $C_S$ and HR | -0.079 | 0.045 | 0.124 | 0.088 | 0.098 | -0.089 |
| *P* value | 0.01 | 0.31 | 0.0008 | $2\times10^{-11}$ | $2\times10^{-7}$ | 0.06 |
| Mean spindle density (pre ECG correction) | 2.31 | 3.01 | 2.26 | 1.56 | 1.48 | 1.52 |
| Mean spindle density (post ECG correction) | 2.35 | 3.11 | 2.38 | 1.81 | 1.55 | 1.57 |
| Mean change in spindle density post ECG correction | 0.04 | 0.10 | 0.12 | 0.25 | 0.07 | 0.05 |
| *t*-statistic (paired pre/post *t*-test) | 12.64 | 20.10 | 28.98 | 74.58 | 47.23 | 14.47 |
| *P* value from *t*-test | $< 10^{-15}$ | $< 10^{-15}$ | $< 10^{-15}$ | $< 10^{-15}$ | $< 10^{-15}$ | $< 10^{-15}$ |

**Supplementary Table 1. EEG/ECG coherence, body mass index, heart rate and spindle density.** For each study, mean spectral coherence for sigma (11-15 Hz) EEG and ECG, along with the body mass index (BMI) mean and its correlation with spectral coherence (similarly, for heart rate, HR). Lower rows show the mean spindle density (spindles per minute) before and after correction for potential cardiac interference, along with tests for a statistically significant change in rate.

| | | Percentiles | |
|---|---|---|---|
| Spindle feature | Mean | 25th | 75th |
| Density (per min) | 1.88 | 1.13 | 2.59 |
| Amplitude (μV) | 30.6 | 19.9 | 36.1 |
| Duration (s) | 0.85 | 0.63 | 0.99 |
| Frequency (Hz) | 13.11 | 12.66 | 13.58 |
| Number of oscillations | 10.9 | 8.0 | 12.5 |
| Symmetry index | 0.49 | 0.38 | 0.61 |

**Supplementary Table 2. Primary spindle properties.** The mean and $25^{th}/75^{th}$ percentiles for the primary individual-mean spindle properties from the canonical $F_C$ = 13.5 Hz analysis.

| | Density | Amplitude | Duration | Frequency | Oscillations | Symmetry |
|---|---|---|---|---|---|---|
| **Density** | . | | | | | |
| **Amplitude** | 0.39 | . | | | | |
| **Duration** | 0.48 | 0.28 | . | | | |
| **Frequency** | 0.25 | -0.06 | -0.10 | . | | |
| **Oscillations** | 0.54 | 0.26 | 0.96 | 0.15 | . | |
| **Symmetry** | 0.13 | 0.00 | -0.04 | 0.53 | 0.10 | . |

**Supplementary Table 3. Partial correlation matrix for spindle properties.** Pairwise partial correlations conditioning on age and sex, calculated in the entire sample for the mean primary spindle density properties per individual, based on the canonical $F_C$ = 13.5 Hz spindle analysis.

| | | | Test/retest correlations | | | |
|---|---|---|---|---|---|---|
| | | | Raw scores | | Age, sex & race corrected | |
| | | N | *R* | *p* | *R* | *p* |
| Spindle density | CHAT | 245 | 0.80 | $< 10^{-15}$ | 0.74 | $< 10^{-15}$ |
| | SHHS | 2597 | 0.84 | $< 10^{-15}$ | 0.81 | $< 10^{-15}$ |
| | MrOS | 958 | 0.77 | $< 10^{-15}$ | 0.76 | $< 10^{-15}$ |
| Spindle amplitude | CHAT | 231 | 0.46 | $2\times10^{-9}$ | 0.40 | $3\times10^{-10}$ |
| | SHHS | 2564 | 0.85 | $< 10^{-15}$ | 0.82 | $< 10^{-15}$ |
| | MrOS | 940 | 0.71 | $< 10^{-15}$ | 0.71 | $< 10^{-15}$ |
| Spindle duration | CHAT | 201 | 0.57 | $< 10^{-15}$ | 0.56 | $< 10^{-15}$ |
| | SHHS | 2567 | 0.72 | $< 10^{-15}$ | 0.67 | $< 10^{-15}$ |
| | MrOS | 938 | 0.54 | $< 10^{-15}$ | 0.54 | $< 10^{-15}$ |
| Spindle frequency | CHAT | 220 | 0.69 | $< 10^{-15}$ | 0.68 | $< 10^{-15}$ |
| | SHHS | 2592 | 0.64 | $< 10^{-15}$ | 0.64 | $< 10^{-15}$ |
| | MrOS | 952 | 0.65 | $< 10^{-15}$ | 0.64 | $< 10^{-15}$ |
| Spindle oscillations | CHAT | 221 | 0.61 | $< 10^{-15}$ | 0.59 | $< 10^{-15}$ |
| | SHHS | 2565 | 0.72 | $< 10^{-15}$ | 0.67 | $< 10^{-15}$ |
| | MrOS | 942 | 0.55 | $< 10^{-15}$ | 0.54 | $< 10^{-15}$ |
| Spindle symmetry | CHAT | 240 | 0.33 | $1\times10^{-7}$ | 0.31 | $9\times10^{-7}$ |
| | SHHS | 2519 | 0.47 | $< 10^{-15}$ | 0.44 | $< 10^{-15}$ |
| | MrOS | 901 | 0.38 | $< 10^{-15}$ | 0.37 | $< 10^{-15}$ |
| N2 epochs | CHAT | 386 | 0.34 | $4\times10^{-12}$ | 0.32 | $1\times10^{-10}$ |
| | SHHS | 2554 | 0.39 | $< 10^{-15}$ | 0.37 | $< 10^{-15}$ |
| | MrOS | 939 | 0.29 | $< 10^{-15}$ | 0.28 | $< 10^{-15}$ |

**Supplementary Table 4. Test/retest correlations for spindle properties.** Test/retest correlations for primary spindle properties in the studies with repeated PSGs, both for raw scores and scores corrected for age, sex and race (regressing each measure on age, sex and race within each study, then estimating the correlation of the residuals between the two time-points).

| | N1 sleep (mins) | | N2 sleep (mins) | | N3 sleep (mins) | | REM sleep (mins) | |
|---|---|---|---|---|---|---|---|---|
| **Predictor** | ***b*** | ***p*** | ***b*** | ***p*** | ***b*** | ***p*** | ***b*** | ***p*** |
| Age (yrs) | 0.05 | $1\times10^{-5}$ | -0.18 | 0.00088 | -0.45 | $< 10^{-15}$ | -0.34 | $< 10^{-15}$ |
| Sex (female) | -3.87 | $< 10^{-15}$ | -10.96 | $< 10^{-15}$ | 25.71 | $< 10^{-15}$ | 1.62 | 0.013 |
| Race (black) | 0.33 | 0.38 | 7.03 | $5\times10^{-5}$ | -14.35 | $< 10^{-15}$ | -0.56 | 0.52 |
| Arousal Index | 0.21 | $< 10^{-15}$ | 0.20 | 0.00068 | -0.83 | $< 10^{-15}$ | -0.66 | $< 10^{-15}$ |
| AHI | -0.01 | 0.22 | 0.18 | $4\times10^{-5}$ | 0.02 | 0.58 | -0.04 | 0.075 |

**Supplementary Table 5. Predictors of the relative time spent in each sleep stage.** The dependent variable was the absolute duration (minutes) of sleep in each stage; one multiple linear regression model was fit for each stage, across all studies, with the following predictors: age, sex, race, arousal index and AHI. Qualitatively similar results were obtained using the relative proportion of each sleep stage instead of duration in minutes, with the exception of the effect of age on the proportion of N2 sleep, which showed a positive association ($b$ = 0.09% N2 per year; $p < 10^{-15}$): that is, older individuals have fewer minutes of N2 sleep, but a greater proportion of their sleep is spent in N2 (instead of N3, which declines with increasing age in both absolute and relative terms).

| | N2 spindle density | | N3 spindle density | | N2 spindle density(*) | |
|---|---|---|---|---|---|---|
| **Sleep stage (minutes)** | ***b*** | ***p*** | ***b*** | ***p*** | ***b*** | ***p*** |
| N1 | 0.01 | 0.95 | -0.07 | 0.66 | 0.15 | 0.36 |
| N2 | 4.38 | $1 \times 10^{-11}$ | 0.44 | 0.54 | 3.66 | $2 \times 10^{-6}$ |
| N3 | -5.22 | $< 10^{-15}$ | -0.53 | 0.27 | -5.40 | $< 10^{-15}$ |
| REM | 1.51 | $3 \times 10^{-6}$ | -0.21 | 0.57 | 1.38 | $6 \times 10^{-4}$ |

**Supplementary Table 6. Spindle density and sleep stage duration.** Predictors of sleep stage, from three separate multiple regressions of stage duration (in minutes) on spindle density in either N2 or N3 sleep, with covariates for age, sex, race, study, arousal index and AHI. Coefficients *b* therefore represent the change in the number of minutes of that sleep stage associated with a unit increase in spindle density (spindles per minute). The third column ("N2 spindle density (*)") represents an analysis in the subset of individuals for whom N3 spindle density estimates were available, making it directly comparable to the N3 analysis (middle columns) in terms of interpreting relative effect sizes. (Some individuals – especially older individuals – did not meet criteria for the minimum post-QC duration of N3 sleep.)

| | N1 | | N2 | | N3 | | REM | |
|---|---|---|---|---|---|---|---|---|
| | ***b*** | ***p*** | ***b*** | ***p*** | ***b*** | ***p*** | ***b*** | ***p*** |
| CHAT | -1.52 | 0.10 | 6.25 | 0.05 | -2.19 | 0.37 | -1.09 | 0.58 |
| CCSHS | -0.14 | 0.84 | 4.88 | 0.18 | -3.47 | 0.15 | -1.33 | 0.51 |
| CFS | 0.53 | 0.26 | 10.78 | $6\times10^{-5}$ | -6.17 | $5\times10^{-4}$ | 2.10 | 0.15 |
| SHHS | -0.01 | 0.97 | 3.71 | $4\times10^{-5}$ | -4.73 | $4\times10^{-13}$ | 1.57 | 0.0004 |
| MrOS | 0.03 | 0.92 | 2.03 | 0.11 | -4.55 | $3\times10^{-9}$ | 1.22 | 0.06 |
| SOF | -0.06 | 0.92 | 7.80 | 0.02 | -10.67 | $5\times10^{-6}$ | 3.12 | 0.05 |
| Age × spindle *p*-value | 0.174 | | 0.915 | | $1\times10^{-6}$ | | $2\times10^{-10}$ | |

**Supplementary Table 7. Age-dependent association between N2 spindle density and sleep macro-architecture.** For each study, we fit four linear regression models of total sleep stage duration (dependent variable) as a function of canonical spindle density during N2, covarying for age, sex, race, arousal index and AHI. A final set of analyses based on all individuals (covarying for study) included an age-by-spindle interaction term (lower row). The significant interaction for N3 sleep indicates that the negative N2 spindle density/N3 association was significantly stronger in older individuals. There was also a positive association between N2 spindle density and duration of REM sleep (see also **Supplementary Table 6**), but only in adults (i.e. a significant age-by-spindle interaction).

| | CHAT | CCSHS | CFS | SHHS | MrOS | SOF |
|---|---|---|---|---|---|---|
| *N* | 448 | 509 | 719 | 5572 | 2762 | 430 |
| Age range | 4-10 | 16-19 | 6-88 | 39-90 | 67-97 | 75-95 |
| *Within-study linear effect of age* | | | | | | |
| Beta (per yr) | 0.165 | -0.005 | -0.018 | -0.034 | -0.034 | -0.061 |
| *t*-statistic | 7.8 | -0.1 | -10.4 | -33.9 | -12.1 | -4.7 |
| *p*-value | $5\times10^{-14}$ | 0.94 | $< 10^{-15}$ | $< 10^{-15}$ | $< 10^{-15}$ | $3\times10^{-6}$ |
| *Inclusion of additional* $age^2$ *term* | | | | | | |
| *p*-value (1 df test) | 0.58 | 0.88 | 0.017 | 0.0001 | 0.88 | 0.62 |
| *Inclusion of additional* $age^x$ *terms for* $x = 2 - 5$ | | | | | | |
| *p*-value (4 df test) | 0.19 | 0.30 | 0.004 | $5\times10^{-5}$ | 0.83 | 0.36 |

**Supplementary Table 8. Within-study association of age and spindle density.** Within each study, we fit a linear model of spindle density from the canonical analysis (dependent variable) on age, sex, race, arousal index and AHI. Consistent with the inverted-U life course trajectory of spindle density (**Supplementary Figure 10**), within CHAT (ages 4-10) there was a significant positive association with age; in contrast, the adult samples showed significant negative associations. In CCSHS, the absence of association with age ($p = 0.94$) was expected due to the restriction of range (most individuals were either 17 or 18 years old). Tests of higher-order age terms (bottom two rows) were significant in CFS and SHHS, which spanned the broadest range of ages. Formal modeling of age trajectories (i.e. **Figure 2**) included higher-order age terms up to the fifth. However, for most analyses across the entire sample and age range, the results did not substantively depend on the choice of linear versus higher-order age corrections, as long as study membership (which is highly correlated with age) was included as a covariate (data not shown). To simplify presentation, we elected to report results from linear age effect models in most cases.

| | | | Test/retest means | | | Matched-pair *t*-test | |
|---|---|---|---|---|---|---|---|
| **Spindle property** | | **N** | **Baseline** | **Follow-up** | **Difference** | ***t*-statistic** | ***p*** |
| Density | CHAT | 245 | 2.35 | 2.42 | 0.07 | 2.3 | 0.023 |
| | SHHS | 2597 | 1.86 | 1.63 | -0.23 | -23.7 | $< 10^{-15}$ |
| | MrOS | 958 | 1.67 | 1.41 | -0.26 | -14.2 | $< 10^{-15}$ |
| Amplitude | CHAT | 231 | 36.01 | 34.62 | -0.92 | -1.1 | 0.25 |
| | SHHS | 2564 | 28.92 | 27.58 | -1.35 | -14.9 | $< 10^{-15}$ |
| | MrOS | 940 | 18.80 | 16.52 | -2.25 | -16.3 | $< 10^{-15}$ |
| Duration | CHAT | 201 | 0.95 | 0.95 | 0.00 | 1.1 | 0.29 |
| | SHHS | 2567 | 0.79 | 0.77 | -0.01 | -15.2 | $< 10^{-15}$ |
| | MrOS | 938 | 0.78 | 0.76 | -0.02 | -9.8 | $< 10^{-15}$ |
| Frequency | CHAT | 220 | 12.59 | 12.59 | 0.01 | 0.5 | 0.62 |
| | SHHS | 2592 | 13.16 | 13.12 | -0.05 | -11.8 | $< 10^{-15}$ |
| | MrOS | 952 | 13.19 | 13.12 | -0.07 | -9.8 | $< 10^{-15}$ |
| Oscillations | CHAT | 221 | 11.69 | 11.71 | 0.04 | 0.8 | 0.42 |
| | SHHS | 2565 | 10.00 | 9.78 | -0.23 | -17.6 | $< 10^{-15}$ |
| | MrOS | 942 | 9.90 | 9.61 | -0.28 | -11.5 | $< 10^{-15}$ |
| Symmetry | CHAT | 240 | 0.47 | 0.47 | 0.00 | 0.8 | 0.42 |
| | SHHS | 2519 | 0.49 | 0.48 | 0.00 | -10.6 | $< 10^{-15}$ |
| | MrOS | 901 | 0.49 | 0.48 | 0.00 | -5.3 | $1\times10^{-7}$ |
| N2 epochs | CHAT | 386 | 194.4 | 216.5 | 21.9 | 6.4 | $5\times10^{-10}$ |
| | SHHS | 2554 | 186.9 | 193.4 | 6.4 | 4.7 | $3\times10^{-6}$ |
| | MrOS | 939 | 150.1 | 175.9 | 25.2 | 8.7 | $< 10^{-15}$ |

**Supplementary Table 9. Longitudinal within-individual analysis of age effects for spindle properties.** For primary spindle properties, within studies that had repeated PSG (CHAT, SHHS and MrOS), we tested for significant mean differences between the two time points (matched pair *t*-tests). Despite a short test/retest interval in CHAT children (around 6 months), we nonetheless observed a nominally significant increase in spindle density ($p = 0.02$), consistent with the cross-sectional analyses in children (**Supplementary Table 8**). In adults (SHHS and MrOS), we saw highly significant but negative changes over time, also consistent with the cross-sectional analyses (**Supplementary Table 8**). The increased effect sizes and significance in the adult samples likely result from their larger sample sizes as well as the greater duration between baseline and follow-up testing. "N2 epochs" reflects the number of artifact-free epochs used in analysis.

| Spindle class | Predictor | *b* | *t*-statistic | *p* |
|---|---|---|---|---|
| Slow spindles ($F_C$ = 11 Hz) | Age (yrs) | -0.012 | -21.8 | $< 10^{-15}$ |
| | Sex (female) | -0.006 | -0.5 | 0.63 |
| | Race (black) | -0.222 | -12.7 | $< 10^{-15}$ |
| Fast spindles ($F_C$ = 15 Hz) | Age (yrs) | -0.027 | -27.0 | $< 10^{-15}$ |
| | Sex (female) | 0.302 | 12.7 | $< 10^{-15}$ |
| | Race (black) | -0.282 | -9.0 | $< 10^{-15}$ |

**Supplementary Table 10. Differential association of sex with fast versus slow spindles**. We fit two models, with a dependent variable of spindle density targeting either fast ($F_C$ = 15 Hz) or slow (11 Hz) spindles regressed on age, sex, race, study, BMI, arousal index and AHI. Whereas fast spindles had higher densities in females compared to males ($p < 10^{-15}$), slow spindles show similar rates between sexes ($p = 0.63$).

| | N3 analysis | | N3 spindle density | | | Dependent variable: spindle density | | | | | |
|---|---|---|---|---|---|---|---|---|---|---|---|
| | | | | | | N3 | | N2 | | N2* | |
| **Study** | ***N* included** | ***N* excluded** | **Mean** | **Ratio to N2** | **Predictors** | ***b*** | ***p*** | ***b*** | ***p*** | ***b*** | ***p*** |
| **CHAT** | 1,061 | 171 | 1.36 | 0.58 | **Age (years)** | 0.08 | 0.005 | 0.16 | $1\times10^{-8}$ | 0.16 | $3\times10^{-8}$ |
| | | | | | **Sex (female)** | 0.21 | 0.002 | 0.28 | $4\times10^{-5}$ | 0.29 | $3\times10^{-5}$ |
| | | | | | **Race (black)** | -0.09 | 0.19 | -0.43 | $3\times10^{-9}$ | -0.43 | $2\times10^{-9}$ |
| **CCSHS** | 502 | 13 | 2.18 | 0.70 | **Age (years)** | -0.03 | 0.77 | 0.05 | 0.47 | 0.06 | 0.38 |
| | | | | | **Sex (female)** | 0.31 | $2\times10^{-5}$ | 0.19 | 0.002 | 0.19 | 0.002 |
| | | | | | **Race (black)** | -0.19 | 0.010 | -0.46 | $5\times10^{-12}$ | -0.45 | $1\times10^{-11}$ |
| **CFS** | 605 | 125 | 1.79 | 0.75 | **Age (years)** | -0.01 | 0.003 | -0.02 | $< 10^{-15}$ | -0.02 | $< 10^{-15}$ |
| | | | | | **Sex (female)** | 0.19 | 0.01 | 0.08 | 0.23 | 0.10 | 0.16 |
| | | | | | **Race (black)** | -0.15 | 0.043 | -0.29 | $2\times10^{-5}$ | -0.29 | $5\times10^{-5}$ |
| **SHHS** | 4,244 | 1,549 | 1.35 | 0.75 | **Age (years)** | -0.02 | $< 10^{-15}$ | -0.03 | $< 10^{-15}$ | -0.04 | $< 10^{-15}$ |
| | | | | | **Sex (female)** | 0.33 | $< 10^{-15}$ | 0.14 | $4\times10^{-9}$ | 0.16 | $1\times10^{-8}$ |
| | | | | | **Race (black)** | -0.08 | 0.11 | -0.25 | $1\times10^{-9}$ | -0.29 | $4\times10^{-8}$ |
| **MrOS** | 1,035 | 1,872 | 1.36 | 0.88 | **Age (years)** | -0.02 | $2\times10^{-5}$ | -0.04 | $< 10^{-15}$ | -0.04 | $2\times10^{-12}$ |
| | | | | | **Sex (female)** | n/a | n/a | n/a | n/a | n/a | n/a |
| | | | | | **Race (black)** | -0.25 | 0.09 | -0.21 | 0.03 | -0.31 | 0.032 |
| **SOF** | 276 | 177 | 1.52 | 0.96 | **Age (years)** | -0.05 | 0.006 | -0.06 | $8\times10^{-5}$ | -0.07 | $6\times10^{-5}$ |
| | | | | | **Sex (female)** | n/a | n/a | n/a | n/a | n/a | n/a |
| | | | | | **Race (black)** | -0.47 | 0.057 | -0.28 | 0.15 | -0.37 | 0.11 |

**Supplementary Table 11. Correlates of N3 and N2 spindles, stratified by study.** Linear regression models of spindle density during either N2 or N3 sleep (dependent variable) regressed on age, sex and race; also, arousal index and AHI were included as covariates in all models. To facilitate comparisons, a third analysis labelled N2* considered N2 spindle density but only in the subset of individuals for whom N3 spindle density was available.

| | | Slow ($F_C$ = 11 Hz) spindles | | | | Fast ($F_C$ = 15 Hz) spindles | | | |
|---|---|---|---|---|---|---|---|---|---|
| | | All N2 | | Persistent N2 | | All N2 | | Persistent N2 | |
| **Predictor** | | ***b*** | ***p*** | ***b*** | ***p*** | ***b*** | ***p*** | ***b*** | ***p*** |
| Sleep cycle number | 1st (ref.) | 0.00 | . | 0.00 | . | 0.00 | . | 0.00 | . |
| | 2nd | -0.81 | $< 10^{-15}$ | -0.69 | $< 10^{-15}$ | -0.85 | $< 10^{-15}$ | -0.62 | $< 10^{-15}$ |
| | 3rd | -1.06 | $< 10^{-15}$ | -0.88 | $< 10^{-15}$ | -0.83 | $< 10^{-15}$ | -0.54 | $< 10^{-15}$ |
| | 4th | -1.18 | $< 10^{-15}$ | -1.00 | $< 10^{-15}$ | -0.64 | $< 10^{-15}$ | -0.34 | $< 10^{-15}$ |
| | 5th | -1.41 | $< 10^{-15}$ | -1.23 | $< 10^{-15}$ | -0.48 | $< 10^{-15}$ | -0.16 | $< 10^{-15}$ |
| Within-cycle position | 0-10 mins (ref.) | 0.00 | . | 0.00 | . | 0.00 | . | 0.00 | . |
| | 10-20 mins | 0.28 | $< 10^{-15}$ | 0.42 | $< 10^{-15}$ | -0.23 | $< 10^{-15}$ | -0.11 | $< 10^{-15}$ |
| | 20-30 mins | 0.17 | $< 10^{-15}$ | 0.26 | $< 10^{-15}$ | -0.52 | $< 10^{-15}$ | -0.44 | $< 10^{-15}$ |
| | 30-40 mins | 0.02 | 0.004 | 0.09 | $< 10^{-15}$ | -0.67 | $< 10^{-15}$ | -0.61 | $< 10^{-15}$ |
| | 40-50 mins | -0.10 | $< 10^{-15}$ | -0.03 | 0.001 | -0.67 | $< 10^{-15}$ | -0.62 | $< 10^{-15}$ |
| | 50-60 mins | -0.20 | $< 10^{-15}$ | -0.11 | $< 10^{-15}$ | -0.63 | $< 10^{-15}$ | -0.57 | $< 10^{-15}$ |
| Local context | Persistent sleep | -0.01 | 0.004 | . | . | -0.46 | $< 10^{-15}$ | . | . |
| | Ascending N2 | -0.28 | $< 10^{-15}$ | -0.29 | $< 10^{-15}$ | -0.34 | $< 10^{-15}$ | -0.23 | $< 10^{-15}$ |
| | Descending N2 | 0.22 | $< 10^{-15}$ | 0.26 | $< 10^{-15}$ | 0.10 | $< 10^{-15}$ | 0.08 | $< 10^{-15}$ |

**Supplementary Table 12. Within-cycle variation in spindle density.** This Table shows results from a series of epoch-level linear mixed models with per-epoch spindle density (either $F_C$ = 11 Hz or 15 Hz) as the dependent variable. All models included epoch-level fixed-effects for sleep cycle number and either a) relative position within the sleep cycle (six-level factor for the first six 10-minute intervals of the cycle), b) ascending/descending status (two binary variables), and c) a binary indicator variable denoting whether that N2 epoch was in persistent sleep or not. A second series of models only included persistent N2 epochs (i.e. and so the coefficient/significance values are given as a period (".") in those cases). All models included a random effect of individual, and fixed individual-level effects of age (including higher-order terms up to the fifth), sex, study and race. Each column shows the regression coefficient (*b*) and significance value (*p*). The results for the sleep cycle number term reported below are from the model that also included within-cycle position. "Ref." indicates that this factor level was set as the reference category.

| Medication | N | *b* | *p* |
|---|---|---|---|
| Benzodiazepines | 305 | 0.20 | $4 \times 10^{-5}$ |
| Tricyclic antidepressants | 166 | -0.21 | 0.0017 |
| Any anti-hypertensive medication | 2312 | -0.07 | 0.0032 |
| Any diuretic | 914 | -0.09 | 0.0034 |
| Nitrates | 154 | -0.15 | 0.024 |
| Inhaled steroids for asthma | 81 | 0.16 | 0.07 |
| Insulins | 91 | -0.15 | 0.08 |
| Oral steroids | 150 | -0.11 | 0.09 |
| Beta-blockers without diuretics | 727 | -0.05 | 0.13 |
| ACE inhibitors without diuretics | 717 | -0.05 | 0.16 |
| Non-steroidal anti-inflammatory agents, excluding aspirin | 1175 | 0.04 | 0.17 |
| Oral hypoglycemic agents | 236 | -0.06 | 0.26 |
| Beta-blockers with diuretics | 32 | -0.16 | 0.27 |
| Any calcium-channel blocker | 814 | -0.03 | 0.30 |
| ACE inhibitors with diuretics | 44 | 0.06 | 0.64 |
| Non-tricyclic antidepressants other than MAOI | 278 | -0.02 | 0.76 |
| Any lipid-lowering medication | 698 | 0.01 | 0.84 |
| Anti-arrhythmics, class 1B | 32 | 0.01 | 0.95 |
| Anti-arrhythmics, class 1A | 24 | 0.00 | 0.98 |

**Supplementary Table 13. Spindle density and medication use in the SHHS.** For SHHS only, results from a series of linear regressions, in which canonical spindle density (dependent variable) was predicted as a function of use of a given medication (binary variable coded 0/1), controlling for age, sex, race, BMI, arousal index and AHI. *N* indicates the number of individuals who reported taking that medication, *b* and *p* are the coefficient (i.e. difference in spindle density) and significance for that term, respectively. Rows are sorted in order of increasing *p*-value.

| Medication | *N* | *b* | *p* |
|---|---|---|---|
| Took zolpidem within 24 hours of PSG | 40 | 0.58 | $1\times10^{-5}$ |
| Zolpidem use | 57 | 0.48 | $1\times10^{-5}$ |
| Nonbenzo nonbarbituate sedative hypnotics | 59 | 0.46 | $3\times10^{-5}$ |
| NSAIDs | 600 | 0.15 | $7\times10^{-5}$ |
| Hypoglycemic agents | 286 | -0.20 | 0.00021 |
| Short acting benzodiazephines | 87 | 0.34 | 0.00021 |
| Benzodiazepines | 133 | 0.27 | 0.00034 |
| Insulin | 52 | -0.38 | 0.0014 |
| Reported taking medications for sleep | 347 | 0.15 | 0.0022 |
| Anticoagulants-coumarin derivatives | 254 | -0.16 | 0.005 |
| Alzheimers disease medications | 51 | -0.28 | 0.019 |
| Cox-II inhibitors | 207 | 0.13 | 0.025 |
| Vitamin D | 1814 | 0.05 | 0.11 |
| Calcium channel blockers | 437 | -0.06 | 0.14 |
| Phosphodiesterase type 5 inhibitors | 63 | 0.16 | 0.14 |
| Loop diuretics | 172 | -0.10 | 0.14 |
| Opiod analgesics | 125 | -0.11 | 0.15 |
| Salicylate (aspirin) use | 1695 | 0.04 | 0.17 |
| Antilepemic: HMG-CoA reductase inhibitors (statin) | 1217 | -0.04 | 0.18 |
| Hypotensive agents-angiotensin II | 278 | -0.07 | 0.21 |
| Erectile dysfunction medications | 65 | 0.13 | 0.21 |
| Antiulcer-proton pump inhibitors | 422 | 0.05 | 0.23 |
| TZD medication (sub of hypoglycemics) | 67 | -0.12 | 0.28 |
| Trazadone use | 41 | -0.14 | 0.29 |
| Long acting benzodiazepines | 48 | 0.13 | 0.30 |
| Anticonvulsants | 96 | 0.09 | 0.31 |
| ACE inhibitors | 734 | -0.03 | 0.43 |
| Antiulcer-H2 antagonists | 197 | -0.05 | 0.43 |
| Alpha-adrenergic blockers | 567 | -0.03 | 0.44 |
| Nitrates | 127 | -0.05 | 0.48 |
| Thiazide diuretics | 538 | 0.03 | 0.52 |
| Potassium-sparing diuretics | 149 | -0.04 | 0.54 |
| Androgen | 42 | 0.08 | 0.55 |
| Gemfibrozil | 48 | -0.06 | 0.60 |
| Bisphosphonates | 123 | 0.03 | 0.67 |
| Osteoporosis drugs | 123 | 0.03 | 0.67 |
| Tricyclic antidepressants | 41 | 0.05 | 0.70 |
| Sildenafil | 55 | 0.04 | 0.70 |
| SSRI antidepressants | 127 | 0.02 | 0.76 |
| Beta blockers, nonopthamalic | 805 | 0.01 | 0.83 |
| Urinary antispasmodic medication | 56 | 0.02 | 0.86 |
| Calcium | 909 | 0.00 | 0.89 |
| Antidepressants | 228 | 0.01 | 0.90 |
| Thyroid agonists | 240 | -0.01 | 0.91 |
| Antiandrogen | 15 | 0.00 | 0.99 |

**Supplementary Table 14. Spindle density and medication use in the MrOS.** Similar to the analyses in **Supplementary Table 13**, but applied to MrOS. In both cases, sedative/hypnotic medications (benzodiazepines and Zolpidem, a nonbenzodiazepine compound but that binds to GABA receptors at the same location as benzodiazepines) were most strongly associated with increased spindle density.

| | AHI | CHAT | CCSHS | CFS | SHHS | MrOS | SOF | Combined |
|---|---|---|---|---|---|---|---|---|
| **Proportion of individuals** | | | | | | | | |
| None | < 5 | 62% | 97% | 53% | 33% | 19% | 23% | 34% |
| Mild | 5 - 15 | 31% | 2% | 22% | 35% | 36% | 39% | 33% |
| Moderate | 15 - 30 | 8% | 1% | 13% | 20% | 27% | 24% | 20% |
| Severe | > 30 | 0% | 0% | 12% | 12% | 18% | 13% | 12% |
| **Spindle density** | | | | | | | | |
| None | < 5 | 2.30 | 3.11 | 2.58 | 1.96 | 1.55 | 1.60 | . |
| Mild | 5 - 15 | 2.38 | 3.19 | 2.29 | 1.82 | 1.61 | 1.65 | . |
| Moderate | 15 - 30 | 2.41 | n/a | 2.37 | 1.67 | 1.54 | 1.52 | . |
| Severe | > 30 | n/a | n/a | 1.70 | 1.61 | 1.43 | 1.38 | . |

**Supplementary Table 15. Mean spindle density by AHI category and study.** The upper panel shows the percentage of individuals in each study within a given AHI range (labeled to denote mild, moderate or severe sleep apnea symptoms). The lower panel shows the average spindle density for each group (or "n/a" if not enough individuals were in that cell).

| | Univariate model | | Joint model | |
|---|---|---|---|---|
| **Predictor of spindle density** | ***b*** | ***p*** | ***b*** | ***p*** |
| AHI | -0.003 | $9\times10^{-10}$ | -0.002 | 0.0002 |
| Arousal Index | -0.004 | $6\times10^{-7}$ | -0.002 | 0.02 |
| log(AHI) | -0.021 | 0.003 | -0.013 | 0.10 |
| log(Arousal Index) | -0.053 | 0.001 | -0.042 | 0.015 |

**Supplementary Table 16. Association of spindles and arousal/apnea indices.** Results from multiple linear regression model of spindle density on either arousal index or AHI ("univariate model"), or both arousal index and AHI jointly ("joint model"), with covariates age, sex, race and study. The lower rows represent the same analyses, but with log-transformed values of arousal index and AHI.

| Sleep stage | Race | CHAT | CCSHS | CFS | SHHS | MrOS | SOF |
|---|---|---|---|---|---|---|---|
| N1 sleep | White | 8.3 | 4.1 | 5.1 | 5.2 | 6.5 | 5.0 |
| | Black | 8.2 | 4.1 | 4.7 | 5.4 | 6.2 | 5.4 |
| N2 sleep | White | 40.2 | 51.8 | 55.5 | 56.4 | 62.7 | 55.6 |
| | Black | 43.1 | 52.1 | 57.7 | 60.0 | 65.1 | 57.9 |
| N3 sleep | White | 32.8 | 24.1 | 21.0 | 18.2 | 11.4 | 20.5 |
| | Black | 30.3 | 21.3 | 18.3 | 14.8 | 7.8 | 17.0 |
| REM | White | 18.5 | 19.8 | 18.3 | 19.8 | 19.2 | 18.5 |
| | Black | 18.3 | 22.1 | 18.7 | 19.7 | 20.6 | 18.8 |

**Supplementary Table 17. Relative duration of sleep stage, stratified by study and race.** Average percentage of sleep scored as N1, N2, N3 or REM in each study, based on manual staging, stratified by race (black and white).

| | White versus black | | Other versus black | |
|---|---|---|---|---|
| | ***b*** | ***p*** | ***b*** | ***p*** |
| **N1 (mins)** | -0.3 | 0.46 | 1.4 | 0.012 |
| **N2 (mins)** | -8.0 | $4\times10^{-6}$ | -5.8 | 0.029 |
| **N3 (mins)** | 13.6 | $< 10^{-15}$ | 9.7 | $1\times10^{-7}$ |
| **REM (mins)** | 0.1 | 0.94 | -0.7 | 0.58 |
| **Sleep efficiency** | 1.36 | 0.0002 | -0.15 | 0.79 |
| **WASO** | -9.59 | $1\times10^{-9}$ | -4.03 | 0.08 |
| **Total sleep time** | 6.88 | 0.0002 | 3.89 | 0.16 |

**Supplementary Table 18. Racial group differences in relative sleep stage duration.** Linear regression models of sleep macro-architecture measures (dependent variables being either stage duration, sleep efficiency, WASO or total sleep time) as a function of race – coded as a three-level factor, black (reference), white and other – and controlling for age, sex, study, BMI, arousal index and AHI.

| | *Multiple logistic regression of race on power plus covariates* | | | *Univariate linear regressions of power on race plus covariates* | | |
|---|---|---|---|---|---|---|
| **Band** | ***OR(black)*** | ***t*-statistic** | ***p*-value** | ***b*** | ***t*-statistic** | ***p*-value** |
| Slow | 1.65 | 6.76 | $1\times10^{-11}$ | 0.20 | 10.13 | $< 10^{-15}$ |
| Delta | 0.74 | -1.79 | 0.074 | -0.13 | -8.63 | $< 10^{-15}$ |
| Theta | 0.52 | -4.00 | $6\times10^{-5}$ | -0.25 | -14.12 | $< 10^{-15}$ |
| Alpha | 1.77 | 4.83 | $1\times10^{-6}$ | -0.33 | -16.30 | $< 10^{-15}$ |
| **Sigma** | **0.24** | **-14.71** | $\mathbf{< 10^{-15}}$ | **-0.46** | **-23.45** | $\mathbf{< 10^{-15}}$ |
| Beta | 1.68 | 4.42 | $1\times10^{-5}$ | -0.24 | -15.12 | $< 10^{-15}$ |

**Supplementary Table 19. Spectral power and racial group differences.** The left panel shows a multiple logistic regression of race (black versus white) on spectral band power estimates averaged over N2 sleep. This analysis represents the partial and independent effect of each band power, although note that some estimates were very highly correlated with each other (e.g. alpha and sigma). The right panel shows the same association framed differently: six linear regressions of band power on race (black versus white). Both models included covariates of age, sex, study, BMI, arousal index and AHI. In both analyses, sigma band (in **bold**) power showed the strongest association with race, having lower values in blacks. Similar results were obtained using relative instead of absolute power (data not shown).

| | *N individuals* | | | Spindle density | | | White vs. black | | Other vs. black | |
|---|---|---|---|---|---|---|---|---|---|---|
| | White | Black | Other | White | Black | Other | *b* | *p* | *b* | *p* |
| CHAT | 485 | 573 | 146 | 2.51 | 2.21 | 2.27 | 0.41 | $7\times10^{-10}$ | 0.00 | 0.98 |
| CCSHS | 307 | 185 | 23 | 3.25 | 2.85 | 3.18 | 0.41 | $5\times10^{-12}$ | 0.35 | 0.010 |
| CFS | 302 | 406 | 22 | 2.49 | 2.28 | 2.69 | 0.30 | $2\times10^{-6}$ | 0.08 | 0.65 |
| SHHS | 4899 | 514 | 380 | 1.81 | 1.57 | 2.16 | 0.26 | $4\times10^{-11}$ | 0.18 | 0.002 |
| MrOS | 2647 | 99 | 161 | 1.55 | 1.44 | 1.59 | 0.21 | 0.016 | 0.20 | 0.057 |
| SOF | 415 | 38 | 0 | 1.57 | 1.55 | n/a | 0.26 | 0.15 | n/a | n/a |
| Combined | . | . | . | . | . | . | 0.284 | $< 10^{-15}$ | 0.215 | $1\times10^{-7}$ |

**Supplementary Table 20. Racial group differences and spindle density.** The left panel gives the number of individuals for each study/race that were included in the analysis of racial differences in spindle density. The next panel gives the mean spindle density for each cell. The final two sets of columns give the regression coefficients and significance values from within-study linear regressions of spindle density on race (coded as a three-level factor with blacks as the reference group), controlling for age, sex, BMI, arousal index and AHI. The bottom row shows the equivalent statistics from an analysis of all individuals, additionally controlling for study.

| Parameter | Study | Group means | | | White vs black | | Other versus black | |
|---|---|---|---|---|---|---|---|---|
| | | White | Black | Other | *b* | *p* | *b* | *p* |
| **Frequency** | CHAT | 12.51 | 12.58 | 12.54 | -0.028 | 0.28 | -0.028 | 0.48 |
| | CCSHS | 13.04 | 13.03 | 13.04 | 0.010 | 0.70 | 0.012 | 0.83 |
| | CFS | 13.19 | 13.16 | 13.10 | 0.024 | 0.23 | 0.020 | 0.74 |
| | SHHS | 13.16 | 13.19 | 13.19 | -0.027 | 0.02 | -0.056 | 0.001 |
| | MrOS | 13.18 | 13.18 | 13.10 | 0.013 | 0.63 | -0.071 | 0.04 |
| | SOF | 13.22 | 13.25 | NA | -0.023 | 0.65 | n/a | n/a |
| | Combined | . | . | . | -0.006 | 0.51 | -0.030 | 0.02 |
| **Duration** | CHAT | 1.00 | 0.95 | 0.96 | 0.039 | $7\times10^{-6}$ | -0.008 | 0.56 |
| | CCSHS | 0.92 | 0.89 | 0.91 | 0.035 | $2\times10^{-6}$ | 0.028 | 0.10 |
| | CFS | 0.84 | 0.84 | 0.89 | 0.009 | 0.09 | 0.013 | 0.41 |
| | SHHS | 0.79 | 0.78 | 0.81 | 0.010 | 0.001 | 0.000 | 0.95 |
| | MrOS | 0.78 | 0.78 | 0.78 | 0.001 | 0.86 | 0.002 | 0.82 |
| | SOF | 0.78 | 0.77 | NA | 0.017 | 0.17 | n/a | n/a |
| | Combined | . | . | . | 0.015 | $5\times10^{-11}$ | 0.006 | 0.0808 |
| **Amplitude** | CHAT | 39.32 | 32.80 | 33.39 | 8.86 | $1\times10^{-11}$ | 4.01 | 0.04 |
| | CCSHS | 37.51 | 25.85 | 32.75 | 11.46 | $< 10^{-15}$ | 6.95 | 0.0003 |
| | CFS | 28.56 | 24.13 | 29.64 | 5.68 | $< 10^{-15}$ | 1.47 | 0.45 |
| | SHHS | 29.10 | 25.04 | 29.55 | 4.49 | $< 10^{-15}$ | 2.29 | 0.0001 |
| | MrOS | 18.27 | 18.41 | 19.60 | -0.14 | 0.83 | 0.92 | 0.24 |
| | SOF | 22.13 | 21.65 | NA | 0.19 | 0.91 | n/a | n/a |
| | Combined | . | . | . | 5.264 | $< 10^{-15}$ | 3.664 | $< 10^{-15}$ |

**Supplementary Table 21. Spindle frequency, duration and amplitude, stratified by study and race**. Similar analyses for those presented in **Supplementary Table 20**, but for spindle frequency, duration and amplitude. Spindle frequency here refers to an individual's mean frequency of observed spindles, from the canonical analysis that targeted $F_C$ = 13.5 Hz.

| | | Regression of spindle density on race, SES and covariates | | | | Group mean for SES | |
|---|---|---|---|---|---|---|---|
| **Study** | **Predictor** | ***b*** | $p_{Race}$ | $p_{SES}$ | $p_{JOINT}$ | **Black** | **White** |
| CCSHS | Race (black) | -0.39 | $4\times10^{-12}$ | . | $4\times10^{-10}$ | 5.85 | 6.68 |
| | SES | 0.01 | . | 0.045 | 0.62 | $p = 6\times10^{-6}$ | |
| CFS | Race (black) | -0.32 | $2\times10^{-6}$ | . | $1\times10^{-6}$ | 5.16 | 5.96 |
| | SES | 0.01 | . | 0.14 | 0.47 | $p = 1\times10^{-7}$ | |
| MrOS | Race (black) | -0.21 | 0.015 | . | 0.016 | 5.15 | 5.94 |
| | Parental education | 0.000 | . | 0.56 | 0.99 | $p = 7\times10^{-5}$ | |
| CHAT | Race (black) | -0.40 | $3\times10^{-10}$ | . | $1\times10^{-7}$ | 2.28 | 3.17 |
| | Parental education | -0.01 | . | 0.069 | 0.78 | $p = 2\times10^{-15}$ | |
| | Race (black) | -0.40 | $3\times10^{-10}$ | . | $3\times10^{-6}$ | 3.80 | 6.59 |
| | Household income | 0.003 | . | 0.004 | 0.85 | $p < 10^{-15}$ | |
| | Race (black) | -0.45 | $3\times10^{-10}$ | . | $5\times10^{-9}$ | 0.46 | 0.11 |
| | Parental unemployment | 0.09 | . | 0.45 | 0.23 | $p = 5\times10^{-12}$ | |
| | Race (black) | -0.47 | . | . | $4\times10^{-6}$ | . | . |
| | Education, income & unemployment | . | . | . | ( 0.22, 0.86 & 0.36 ) | | |

**Supplementary Table 22. Testing for a mediating role of socio-economic status in the spindle/race association.** For the four studies with available measures of socio-economic status (SES), we compared three models in which (canonical) spindle density was predicted by either 1) race (black versus white), 2) SES or 3) race and SES (*joint*). All analyses included covariates for age, sex (where appropriate), BMI, arousal index and AHI. The column of regression coefficients (*b*) is taken from the joint race/SES model. The rightmost set of columns shows group means for SES by race (black versus white) and a *t*-test for a group difference. Household income in CHAT was coded on an 8-point scale. In all cases we observed significant differences between blacks and whites for the SES measures, as well as for spindle density ($p_{Race}$). However, the association between SES and spindle density was typically modest or absent ($p_{SES}$). Furthermore, when controlling for SES, the association between spindle density and race remained in all instances, whereas terms for SES were all $p > 0.05$ in the joint model ($p_{Joint}$). This suggests that although these particular measures of SES exhibited racial differences, they did not account for the observed racial differences in spindle density.

| | MZ Twin Pairs | | Unrelated pairs | |
|---|---|---|---|---|
| *First pair* | | | | |
| Age | 11 | 11 | 11 | 11 |
| Sex | F | F | F | F |
| Race | B | B | B | B |
| Spindle density | 3.47 | 3.74 | 2.75 | 2.46 |
| Spindle amplitude | 36.3 | 36.2 | 55.4 | 33.0 |
| Spindle duration | 0.99 | 0.95 | 0.98 | 0.83 |
| Spindle frequency | 12.94 | 12.74 | 12.85 | 12.75 |
| *Second pair* | | | | |
| Age | 51 | 51 | 51 | 51 |
| Sex | M | M | M | M |
| Race | W | W | W | W |
| Spindle density | 2.59 | 2.54 | 1.83 | 3.23 |
| Spindle amplitude | 14.7 | 18.4 | 20.8 | 32.2 |
| Spindle duration | 0.80 | 0.73 | 0.75 | 0.76 |
| Spindle frequency | 13.61 | 13.65 | 12.55 | 13.19 |

**Supplementary Table 23. Demographic and spindle properties for the two illustrative MZ pairs and matched controls.** Spindle properties were from the canonical analysis (targeting 13.5 Hz spindles). All pairs were selected from the CFS. Units: spindle density is in spindles per minute; amplitude is in μV units; duration is in seconds; frequency is in Hz.

| Phenotype | ICC | 95% confidence interval |
|---|---|---|
| Slow | 0.20 | ( 0.09 - 0.32 ) |
| Delta | 0.33 | ( 0.22 - 0.44 ) |
| Theta | 0.32 | ( 0.21 - 0.43 ) |
| Alpha | 0.33 | ( 0.22 - 0.44 ) |
| Sigma | 0.39 | ( 0.28 - 0.50 ) |
| Beta | 0.29 | ( 0.17 - 0.40 ) |
| Spindle density | 0.34 | ( 0.23 - 0.45 ) |
| Spindle amplitude | 0.42 | ( 0.31 - 0.52 ) |
| Spindle duration | 0.25 | ( 0.13 - 0.36 ) |
| Spindle frequency | 0.35 | ( 0.24 - 0.46 ) |
| Spindle oscillations | 0.23 | ( 0.12 - 0.35 ) |
| Spindle symmetry | 0.22 | ( 0.11 - 0.34 ) |

**Supplementary Table 24. Sibling intraclass correlations for spectral and spindle traits.** In the CFS, we calculated intraclass correlation (ICC) coefficients to estimate the variance explained by familial factors (shared genetics and shared environment). Because age and race tend to be obligatorily similar for full siblings, measures were first adjusted for the effects of age, race and also sex: ICCs were calculated on the residuals from a linear regression model in which the above measures were predictors. Sibship size ($S$) for $S = 2, 3, \ldots, 8$ where 46, 11, 8, 3, 1, 0, 1 for whites and 56, 22, 10, 1, 1, 0, 0 for blacks.

| | Genetic correlations | | | | Phenotypic correlations | | | |
|---|---|---|---|---|---|---|---|---|
| | White CFS families | | Black CFS families | | White CFS families | | Black CFS families | |
| | $r_G$ | $p$ | $r_G$ | $p$ | $r$ | $p$ | $r$ | $p$ |
| **Sleep stage duration (mins)** | | | | | | | | |
| N1 sleep | -0.06 | 0.41 | -0.13 | 0.27 | 0.04 | 0.54 | 0.00 | 0.93 |
| N2 sleep | **0.62** | **0.04** | **0.93** | **0.03** | **0.15** | **0.01** | **0.10** | **0.04** |
| N3 sleep | **-0.45** | **0.03** | **-0.48** | **0.04** | -0.10 | 0.10 | -0.06 | 0.23 |
| REM sleep | 0.13 | 0.32 | -0.17 | 0.27 | **0.13** | **0.03** | 0.06 | 0.27 |
| Total sleep time | 0.15 | 0.30 | -0.13 | 0.32 | 0.04 | 0.49 | 0.04 | 0.42 |
| **Spectral band power** | | | | | | | | |
| Slow | **-0.50** | **0.03** | -0.39 | 0.09 | **-0.22** | **0.0001** | -0.02 | 0.74 |
| Delta | **-0.58** | **0.02** | **-0.42** | **0.04** | **-0.24** | **$4\times10^{-5}$** | -0.07 | 0.17 |
| Theta | **-0.61** | **0.02** | -0.25 | 0.13 | **-0.30** | **$2\times10^{-7}$** | **-0.14** | **0.004** |
| Alpha | -0.01 | 0.48 | -0.24 | 0.14 | **-0.18** | **0.002** | -0.10 | 0.06 |
| Sigma | 0.23 | 0.10 | **0.44** | **0.01** | **0.30** | **$1\times10^{-7}$** | **0.39** | **$< 10^{-15}$** |
| Beta | 0.05 | 0.42 | 0.01 | 0.48 | **-0.15** | **0.01** | **-0.14** | **0.004** |
| **Spindle phenotypes** | | | | | | | | |
| Amplitude | **0.49** | **0.01** | **0.43** | **0.02** | **0.36** | **$1\times10^{-10}$** | **0.39** | **$4\times10^{-16}$** |
| Duration | **0.61** | **0.004** | 0.35 | 0.11 | **0.43** | **$1\times10^{-14}$** | **0.55** | **$< 10^{-15}$** |
| Frequency | 0.13 | 0.29 | 0.02 | 0.47 | **0.15** | **0.01** | **0.18** | **0.0003** |
| Oscillations | **0.64** | **0.003** | 0.33 | 0.13 | **0.46** | **$< 10^{-15}$** | **0.57** | **$< 10^{-15}$** |
| Symmetry | 0.17 | 0.26 | 0.26 | 0.23 | **0.14** | **0.02** | 0.08 | 0.13 |
| N3 density | **0.89** | **0.0002** | **0.88** | **$1\times10^{-5}$** | **0.75** | **$< 10^{-15}$** | **0.80** | **$< 10^{-15}$** |

**Supplementary Table 25. Genetic correlations between spindle density and other sleep, spectral and spindle traits.** Separately for black and white CFS individuals, we estimated the phenotypic correlations (i.e. cross-trait, within-individual, directly from the observed data) and genetic correlations $r_G$ (estimates of the extent of overlap in genetic influences for two traits, see Methods). Note that heritable traits that are phenotypically uncorrelated are unlikely to show significant genetic overlap – although highly correlated traits could be genetically unrelated, if shared environmental factors induce the association. Power to detect significant genetic correlations is lower than for univariate heritabilities: the point estimates are likely to fluctuate and non-significant results can reflect low statistical power. Significant correlations ($p < 0.05$) are in bold.

| | White CFS families | | Black CFS families | |
|---|---|---|---|---|
| | ***b*** | ***p*** | ***b*** | ***p*** |
| Slow spindles (11Hz) ($h^2$) | 0.21 | 0.02 | 0.40 | $5\times10^{-6}$ |
| Fast spindles (15Hz) ($h^2$) | 0.40 | $7\times10^{-5}$ | 0.49 | $5\times10^{-8}$ |
| Fast/slow spindle genetic correlation ($r_G$) | -0.15 | 0.31 | 0.16 | 0.18 |

**Supplementary Table 26. Heritabilities and genetic correlation for fast and slow spindle densities.** Separately for black and white CFS individuals, we estimated univariate heritabilities and genetic correlations for fast (targeting 15 Hz) and slow (targeting 11 Hz) spindle densities. Although both spindle measures showed significant heritability, there was no evidence for genetic overlap as measured by the genetic correlation.

| | Sigma EEG coherence | | Index of spindle bilaterality | | | | | | |
|---|---|---|---|---|---|---|---|---|---|
| | | | | Spindle density | | Age (yrs) | | Sex (female) | |
| **Study** | **Mean** | ***r*( coh, spindles )** | **Mean** | ***b*** | ***p*** | ***b*** | ***p*** | ***b*** | ***p*** |
| CHAT | 0.33 | 0.28 | 0.56 | 0.074 | $< 10^{-15}$ | -0.002 | 0.60 | 0.007 | 0.40 |
| CCSHS | 0.42 | 0.15 | 0.57 | 0.063 | $< 10^{-15}$ | 0.019 | 0.029 | -0.003 | 0.68 |
| CFS | 0.36 | 0.30 | 0.52 | 0.088 | $< 10^{-15}$ | -0.001 | 0.019 | 0.010 | 0.17 |
| SHHS | 0.12 | 0.45 | 0.43 | 0.130 | $< 10^{-15}$ | -0.001 | $< 10^{-15}$ | 0.006 | 0.03 |
| MrOS | 0.28 | 0.36 | 0.44 | 0.125 | $< 10^{-15}$ | -0.001 | 0.07 | n/a | n/a |
| SOF | 0.23 | 0.29 | 0.42 | 0.118 | $< 10^{-15}$ | -0.001 | 0.38 | n/a | n/a |
| Combined | . | . | . | 0.122 | $< 10^{-15}$ | -0.001 | $< 10^{-15}$ | 0.005 | 0.046 |

**Supplementary Table 27. Inter-hemispheric sigma-band coherence and spindle bilaterality.** Mean sigma (11-15 Hz) spectral inter-hemispheric coherence in the EEG (between C3 and C4), calculated within study, and the correlation with spindle density (all highly significantly greater than 0). The right group of columns relates to a derived spindle bilaterality measure, calculated per individual as the proportion of spindles concordantly detected at both C3 and C4. Concordance was defined as any overlap between spindles, allowing for a 0.5 sec window around each event (i.e. spindles up to 1 second apart were considered to be bilateral events). Under this definition, approximately half of all spindles were bilateral, with the proportion decreasing with increasing age, as reflected by the study means as well as regressions of bilaterality on age, spindle density, spindle amplitude and sex. Higher spindle density also predicted greater bilaterality, which is consistent with greater chance overlap occurring in individuals with more spindles driving at least part of this effect (similarly for increased amplitude, and therefore detectability).

| Study | Mean spindle density | | Correlation |
|---|---|---|---|
| | Wavelet | Bandpass | |
| CHAT | 2.35 | 3.07 | 0.78 |
| CCSHS | 3.11 | 3.38 | 0.73 |
| CFS | 2.38 | 3.18 | 0.86 |
| SHHS | 1.81 | 3.06 | 0.88 |
| MrOS | 1.55 | 2.61 | 0.91 |
| SOF | 1.57 | 2.95 | 0.89 |
| Combined | 1.88 | 2.97 | 0.83 |

**Supplementary Table 28. Mean spindle density estimates by wavelet and bandpass methods, stratified by study.** We used an alternate method to detect spindles based on bandpass filtering the EEG signal in the sigma range. This table shows study-specific means, compared to the canonical wavelet analysis. The bandpass method yielded higher estimates of spindle density overall (combined sample mean of 2.97 per individual, compared to 1.88). Mean differences between methods will arise due to differences in sensitive and specificity, and by themselves typically do not indicate which, if any, method is preferable. Importantly, the correlations in spindle density estimates from the two methods were very high ($r$ ~ 0.8 – 0.9), which means that for many purposes (e.g. as a phenotype in molecular genetic studies) estimates from either method will be largely equivalent. Similarly, we did not observe substantively different results in any of our primary analyses when using spindle density estimates from the bandpass filter method compared to wavelets (**Supplementary Table 29**).

| Metric | Study | Wavelet | Bandpass |
|---|---|---|---|
| Association with age | Combined | $b$ = -0.031 ($t$ = -37.3) | $b$ = -0.0096 ($t$ = -29.9 ) |
| Association with sex | Combined | $b$ = 0.16 ($t$ = 8.5 ) | $b$ = 0.048 ( $t$ = 6.4 ) |
| Association with race | Combined | $b$ = 0.28 ($t$ = 10.8) | $b$ = 0.09 ($t$ = 8.4) |
| Test/retest correlation | CHAT | 0.80 | 0.61 |
| Test/retest correlation | SHHS | 0.85 | 0.79 |
| Test/retest correlation | MrOS | 0.78 | 0.73 |
| Sibling correlation | CFS | 0.45 ( 0.34 - 0.54 ) | 0.40 ( 0.29 - 0.50 ) |
| Sibling correlation, adjusted for age/sex effects | CFS | 0.34 ( 0.23 - 0.45 ) | 0.3 ( 0.19 - 0.42 ) |

**Supplementary Table 29. Association with demographic factors, test/retest reliability and sibling correlations for wavelet and bandpass spindle detectors.** In a regression of spindle density (either from the canonical wavelet analysis or the bandpass analysis) as the dependent variable, we observed qualitatively similar results for age, sex and race, although effect sizes (as indirectly indicated by the *t* statistics) were typically greater for the wavelet method. The wavelet method also yielded higher test/retest correlation coefficients and slightly higher sibling intraclass correlations, suggesting it is perhaps a more reliable measure. In broad terms, however, both approaches perform similarly.

| | **Test/retest correlations** | | | **Mean spindle density** | | |
|---|---|---|---|---|---|---|
| $F_C$ | $T_L$ | $T_A$ | **Default** | $T_L$ | $T_A$ | **Default** |
| 8 | 0.31 | 0.41 | 0.46 | 2.49 | 9.16 | 2.17 |
| 9 | 0.31 | 0.39 | 0.48 | 1.93 | 9.03 | 2.18 |
| 10 | 0.35 | 0.36 | 0.54 | 1.47 | 8.71 | 2.19 |
| 11 | 0.41 | 0.36 | 0.71 | 1.13 | 8.42 | 2.27 |
| 12 | 0.40 | 0.36 | 0.77 | 0.90 | 8.28 | 2.41 |
| 13 | 0.40 | 0.41 | 0.75 | 0.71 | 8.21 | 2.70 |
| 14 | 0.37 | 0.51 | 0.72 | 0.56 | 8.15 | 2.84 |
| 15 | 0.30 | 0.55 | 0.72 | 0.46 | 7.75 | 2.33 |
| 16 | 0.23 | 0.47 | 0.68 | 0.40 | 6.93 | 1.36 |
| 17 | 0.16 | 0.29 | 0.50 | 0.42 | 6.54 | 0.72 |
| 18 | 0.09 | 0.24 | 0.33 | 0.43 | 6.50 | 0.52 |

**Supplementary Table 30. Varying spindle detection thresholds and the test/retest reliability of spindle density estimates.** For the different detection thresholds, this table shows the mean test/retest correlations, averaged over all CHAT, SHHS and MrOS individuals, for a range of targeted spindle frequencies. Alternate thresholds are $T_L$ (low-amplitude spindles, between 1 and 2 times the mean) and $T_A$ (all spindles greater than 1 times). Spindle density estimates based on alternate detection thresholds tend to be less reliable, as indexed by the test/retest correlation. The right panel shows the corresponding spindle density means. The more liberal detection threshold tended to show greater rates of slower (<10 Hz) activity.

Note that for the default analysis (with threshold $t = 4.5$) we reported a mean spindle density of 1.88 in the main text. Here, for a $F_C$=13Hz analysis, the estimate is 2.7 however. The difference is driven by the bandwidth (i.e. number of cycles for the wavelet) used in the frequency-dependent analyses versus the canonical analysis (12 versus 7, as we noted in the Methods section). We increased the number of cycles in the frequency-dependent analyses to provide greater frequency resolution, at the expense of temporal resolution. (Note: the difference between $F_C$ of 13 Hz versus 13.5 Hz does not have a great impact here.) The correlation between these two estimates is still very high however, at $r \sim 0.9$. On face value, it may seem counter-intuitive that the broader canonical analysis captures fewer spindles than one more narrowly focused around the target frequency. However, it is important to note that wavelet parameters will impact the baseline distribution of wavelet coefficients as well as the when targeted spindles are actually present. As such, both signal and noise components will be influenced by parameter selection: the resulting impact on sensitivity and specificity may not be straightforwardly predictable.

| | $T_L$ | | $T_A$ | | Default detection thresholds | | | |
|---|---|---|---|---|---|---|---|---|
| | Spindle density | | Spindle density | | Spindle density | | Spindle amplitude | |
| $F_C$ | *b* | *p* | *b* | *p* | *b* | *p* | *b* | *p* |
| 8 | 0.006 | 0.83 | -0.11 | 0.04 | -0.006 | 0.78 | 2.55 | $< 10^{-15}$ |
| 9 | -0.004 | 0.87 | -0.03 | 0.61 | -0.015 | 0.51 | 2.32 | $< 10^{-15}$ |
| 10 | -0.039 | 0.07 | 0.01 | 0.84 | 0.047 | 0.06 | 2.50 | $< 10^{-15}$ |
| 11 | -0.031 | 0.12 | 0.19 | $5\times10^{-5}$ | 0.222 | $3\times10^{-12}$ | 3.37 | $< 10^{-15}$ |
| 12 | -0.077 | $2\times10^{-5}$ | 0.27 | $3\times10^{-9}$ | 0.260 | $4\times10^{-10}$ | 4.22 | $< 10^{-15}$ |
| 13 | -0.033 | 0.053 | 0.32 | $7\times10^{-11}$ | 0.219 | $2\times10^{-5}$ | 5.10 | $< 10^{-15}$ |
| 14 | 0.004 | 0.79 | 0.42 | $< 10^{-15}$ | 0.197 | 0.0005 | 4.75 | $< 10^{-15}$ |
| 15 | -0.008 | 0.53 | 0.29 | $8\times10^{-9}$ | 0.162 | 0.006 | 3.90 | $< 10^{-15}$ |
| 16 | -0.026 | 0.03 | 0.06 | 0.17 | 0.033 | 0.47 | 3.23 | $< 10^{-15}$ |
| 17 | -0.031 | 0.003 | -0.06 | 0.12 | -0.012 | 0.58 | 1.73 | $1\times10^{-7}$ |
| 18 | -0.013 | 0.22 | 0.01 | 0.80 | -0.003 | 0.82 | 1.43 | $2\times10^{-6}$ |

**Supplementary Table 31. Varying spindle detection thresholds and the effect of benzodiazepines on spindle density.** This table shows a series of regressions of spindle density (for a given target frequency and detection threshold) on benzodiazepine use in the SHHS, controlling for age, sex, race, BMI, arousal index and AHI. Estimates of spindle density based on the lower detection threshold ($T_L$) (which assays low-amplitude spindles) showed significant but *negative* relationships with benzodiazepine use, which is counter to the positive relationship observed with typical spindles (see **Supplementary Figure 20** and **Supplementary Table 13**). Furthermore, in the default analysis, spindle amplitude showed an even stronger positive relationship with benzodiazepine use than spindle density (rightmost set of columns). This pattern of results is consistent with benzodiazepines having a primary, or at least an independent, effect on spindle amplitude rather than spindle density (see **Supplementary Figure 28**).

| | $T_L$ | | $T_A$ | | Default | |
|---|---|---|---|---|---|---|
| **Predictor** | ***b*** | ***p*** | ***b*** | ***p*** | ***b*** | ***p*** |
| Age (SD units) | **0.16** | **$10^{-117}$** | **0.09** | **$10^{-6}$** | **-0.61** | **$10^{-204}$** |
| Sex (female) | **-0.04** | **$10^{-8}$** | **-0.26** | **$10^{-38}$** | **0.07** | **$10^{-4}$** |
| Race (other vs. black) | -0.03 | 0.03 | -0.002 | 0.98 | **0.23** | **$10^{-8}$** |
| Race (white vs. black) | **-0.06** | **$10^{-10}$** | -0.023 | 0.41 | **0.25** | **$10^{-21}$** |
| | | | | | | |
| BMI (SD units) | -0.008 | 0.02 | 0.02 | 0.02 | 0.02 | 0.01 |
| Arousal index (SD units) | -0.0001 | 0.97 | -0.03 | 0.001 | -0.02 | 0.12 |
| AHI (SD units) | 0.003 | 0.42 | **-0.05** | **$10^{-6}$** | **-0.04** | **$10^{-4}$** |

**Supplementary Table 32. Varying spindle detection thresholds and demographic associations of spindle density.** The table shows regression coefficients (*b*) and significance values (*p*) for predictors of spindle density (dependent variable) in a series of multiple linear regression models, which vary by spindle detection threshold ($T_L$, $T_A$ and the default analysis). Quantitative variables are standardized, so the *b* indicates change in spindle density per standard deviation (SD) unit of the predictor. Values $p < 0.001$ are **bolded**. Here we do not truncate p-values at $10^{-15}$, in order to indicate relative effect sizes, as all analyses were based on the same set of individuals. Consistent with the effect of benzodiazepines on low-amplitude spindles ($T_L$) in **Supplementary Table 31**, we saw a significant but negative pattern of associations for age, sex and race compared to the default analysis. That is, low-amplitude spindles showed a greater density in older adults, in males and in blacks. **Supplementary Figure 27** plots the corresponding life course trajectories for $T_L$, $T_A$ and default spindles also. This pattern of results is consistent with the effects of age, sex and race being primarily on spindle amplitude, rather than spindle density *per se* (see **Supplementary Figure 28**).

| | | Univariate | Joint models | | |
|---|---|---|---|---|---|
| **Spindle model** | **Predictor(s)** | ***p*** | ***p*** | ***p*** | ***p*** |
| Default analysis, $F_C$ = 13 Hz | Spindle density | $1\times10^{-23}$ | 0.09 | 0.09 | 0.03 |
| | Spindle amplitude | $2\times10^{-73}$ | $1\times10^{-54}$ | $8\times10^{-17}$ | $4\times10^{-43}$ |
| | Spindle duration | $9\times10^{-5}$ | 0.07 | 0.05 | 0.04 |
| | Sigma power | $2\times10^{-66}$ | . | 0.15 | . |
| | Relative sigma power | $2\times10^{-35}$ | . | . | 0.24 |
| $T_A$ analysis, $F_C$ = 13 Hz | Spindle density | 0.50 | 0.01 | 0.01 | 0.01 |
| | Spindle amplitude | $7\times10^{-72}$ | $1\times10^{-71}$ | $7\times10^{-17}$ | $2\times10^{-45}$ |
| | Spindle duration | 0.045 | $4\times10^{-5}$ | $3\times10^{-5}$ | $2\times10^{-5}$ |
| | Sigma power | . | . | 0.83 | . |
| | Relative sigma power | . | . | . | 0.45 |

**Supplementary Table 33. Logistic regression models of race, spindle and spectral traits.** In an attempt to untangle the associations between race and the various spectral and spindle traits, we fit a joint logistic regression model for race (dependent variable, 1=black, 0=white) as a function of spindle density, amplitude and duration, and sigma power (either relative or absolute). All models contained covariates for age, study, sex, BMI, arousal index and AHI. The three joint models (three columns) are defined by the predictors included, a period "." instead of a *p*-value meaning that term was not in the model. To illustrate the impact of detection threshold, analyses were repeated for the default as well as the broader $T_A$ analysis, which included lower amplitude spindles (both with $F_C$ set to 13 Hz; similar results were obtained for other target frequencies). **Default spindle set:** The leftmost column shows the *p*-values for each predictor when entered individually: all terms were significant, as previously indicated (**Supplementary Tables 19, 20 & 21**). The right panel shows three joint models, that included all three spindle traits, along with sigma power (either as an absolute or relative value). Only spindle amplitude had a significant independent effect when entered in the joint model, as shown in the second column. Note that mean sigma power was very highly correlated with mean spindle amplitude: as these terms become almost collinear, resolving their independent effects becomes difficult. With that caveat, the joint models that also included average sigma power (across all N2) still pointed to spindle amplitude as the only significant factor independently associated with race. **Broader $T_A$ spindle set:** Qualitatively similar results were obtained, with the exception that even in the univariate models, spindle amplitude is the only highly significant predictor of race. This is consistent with less confounding between spindle density and amplitude due to the lower detection threshold (i.e. because the probability of detection is less coupled with the true spindle amplitude). Along with the results presented in **Supplementary Tables 31** and **32** and **Supplementary Figure 27**, this suggests that spindle amplitude rather than spindle density *per se* may be a more straightforwardly interpretable metric in these instances (see **Supplementary Figure 28**).

## SUPPLEMENTARY METHODS

### DESCRIPTIONS OF THE NATIONAL SLEEP RESEARCH RESOURCE STUDIES

All data were obtained from the National Sleep Research Resource (NSRR; http://sleepdata.org), a web portal funded by the National Heart, Lung and Blood Institute (NHLBI) to offer free access to large collections of de-identified physiological signals and clinical data elements collected in well-characterized research cohorts and clinical trials. Physiological signals from overnight sleep studies are available as downloadable EDF (European Data Format, [1]) polysomnograms (PSGs), as are standard (Rechtschaffen and Kales or AASM) annotations of these PSGs. All data were collected as part of research protocols.

**Childhood Adenotonsillectomy Trial**

The Childhood Adenotonsillectomy Trial (CHAT) [2], [3] is a multi-center, single-blind, randomized, controlled trial designed to test whether after a 7-month observation period, children, ages 5 to 9.9 years, with mild to moderate obstructive sleep apnea randomized to early adenotonsillectomy (eAT) will show greater levels of neurocognitive functioning, specifically in the attention-executive functioning domain, than children randomized to watchful waiting plus supportive care (WWSC). Other outcomes assessed included other indices of neurocognitive functioning (learning and memory, information processing, etc.), physical growth, blood pressure, metabolic profile, symptoms and quality of life. Physiological measures of sleep were assessed at baseline and at 7-months with standardized full in-laboratory polysomnography with central scoring at the Brigham and Women's Sleep Reading Center. In total, 1,447 children had screening PSGs and 464 were randomized to treatment.

**Cleveland Children's Sleep and Health Study**

The Cleveland Children's Sleep and Health Study (CCSHS) [4], [5] is a population-based pediatric cohort with sleep studies. The cohort includes 907 children, initially studied at ages 8-11 years with in-home sleep studies, acoustic reflectometry, anthropometry, spirometry, blood pressure, and neuropschology and behavioral assessments. The cohort is a stratified random sample of full-term and preterm children, born between 1988-1993, identified from the birth records of 3 Cleveland area hospitals. Data used in the current analyses were from an examination conducted between 2006-2010, when 517 of the children, then between 16 to 19 years of age, participated in a research examination including full in-laboratory polysomnography, home actigraphy, and measurement of demographic, anthropometric and cardiovascular risk factor data.

**Cleveland Family Study**

The Cleveland Family Study (CFS) [6] is a family-based longitudinal study designed to identify the familial basis of sleep apnea, initiated in 1990 and continued through 2006. The entire cohort consists of 2,284 individuals (46% African American) from 361 families studied on up to four occasions over a period of 16 years. Individuals were recruited as probands with diagnosed sleep apnea, neighborhood controls, and the first and selective second-degree relatives of these individuals. Data are based on the last examination (2000-2006), which occurred in a Clinical Research Center, and included full polysomnography, anthropometry, and collection of medical and cardiovascular risk factor information. Sleep data were collected using 14-channel overnight polysomnography (Compumedics E series) in a clinical research unit.

**Sleep Heart Health Study**

The Sleep Heart Health Study (SHHS) [7], [8] is a multi-center cohort study implemented by the National Heart Lung & Blood Institute to determine the cardiovascular and other consequences of

sleep-disordered breathing. It tests whether sleep-related breathing is associated with an increased risk of coronary heart disease, stroke, all cause mortality, and hypertension. In all, 6,441 men and women aged 40 years and older were enrolled between November 1, 1995 and January 31, 1998 and followed with a second examination, approximately 5 to 6 years later (between January 2001- June 2003, SHHS-2), in which 3,295 individuals participated. Polysomnography was performed at home or other unattended settings, using the Compumedics PS-2 system (Compumedics Pty Ltd, Abbotsford, Australia) to record electroencephalgram, chin electromyogram, electro-oculogram, chest and abdominal respiratory excursions, nasal and oral airflow, arterial oxyhemoglobin saturation, a bipolar electrocardiogram, body position, and ambient light. Sleep data were scored at a central Reading Center, using methods for scoring of sleep stages, arousals, and respiratory events detailed elsewhere [8].

**Outcomes of Sleep Disorders In Older Men Study - MrOS-Sleep**

MrOS-Sleep [9], [10] is an ancillary study of the parent Osteoporotic Fractures in Men Study (MrOS). The objectives of the Sleep Study are to understand the relationship between sleep disorders and falls, fractures, mortality, and vascular disease. Between 2000 and 2002, 5,994 community-dwelling men 65 years or older were enrolled at 6 clinical centers in a baseline examination. Between December 2003 and March 2005, 3,135 of these participants were recruited to the Sleep Study when they underwent full, unattended polysomnography and 3 to 5-day actigraphy studies.

**Study of Osteoporotic Fractures**

The Study of Osteoporotic Fractures (SOF) [11] is a multisite, prospective, observational study of community-dwelling women age 65 years and older. The original cohort consisted of 9,704 Caucasian participants residing in four metropolitan areas (Baltimore, MD; Minneapolis, MN; Portland, OR; Monongahela Valley, PA). Initial enrollment took place between September 1986 and October 1988, and participants were re-assessed at biannual follow-up visits. The study was expanded by the addition of 662 African-American women recruited February 1997 to February 1998. Unattended overnight 12 channel in-home polysomnography was completed in a convenience subset of 461 women recruited from two of the four clinical centers (Minnesota and Pittsburgh) in Exam 8 (2002-2004) when they were a mean 82.8 years old.

## Supplementary References


[1] B. Kemp, A. Värri, A. C. Rosa, K. D. Nielsen, and J. Gade, "A simple format for exchange of digitized polygraphic recordings," *Electroencephalogr. Clin. Neurophysiol.*, vol. 82, no. 5, pp. 391–393, 1992.

[2] S. Redline *et al.*, "The Childhood Adenotonsillectomy Trial (CHAT): rationale, design, and challenges of a randomized controlled trial evaluating a standard surgical procedure in a pediatric population," *Sleep*, vol. 34, no. 11, pp. 1509–1517, Nov. 2011.

[3] C. L. Marcus *et al.*, "A randomized trial of adenotonsillectomy for childhood sleep apnea," *N. Engl. J. Med.*, vol. 368, no. 25, pp. 2366–2376, Jun. 2013.

[4] C. L. Rosen *et al.*, "Prevalence and risk factors for sleep-disordered breathing in 8- to 11-year-old children: association with race and prematurity," *J. Pediatr.*, vol. 142, no. 4, pp. 383–389, Apr. 2003.

[5] A. M. Hibbs, A. Storfer-Isser, C. Rosen, C. E. Ievers-Landis, E. M. Taveras, and S. Redline, "Advanced sleep phase in adolescents born preterm," *Behav. Sleep. Med.*, vol. 12, no. 5, pp. 412–424, Sep. 2014.

[6] S. Redline *et al.*, "The familial aggregation of obstructive sleep apnea," *Am. J. Respir. Crit. Care Med.*, vol. 151, no. 3 Pt 1, pp. 682–687, Mar. 1995.

[7] S. F. Quan *et al.*, "The Sleep Heart Health Study: design, rationale, and methods," *Sleep*, vol. 20, no. 12, pp. 1077–1085, Dec. 1997.

[8] S. Redline *et al.*, "Methods for obtaining and analyzing unattended polysomnography data for a multicenter study. Sleep Heart Health Research Group," *Sleep*, vol. 21, no. 7, pp. 759–767, Nov. 1998.

[9] E. Orwoll *et al.*, "Design and baseline characteristics of the osteoporotic fractures in men (MrOS) study — A large observational study of the determinants of fracture in older men," *Contemp. Clin. Trials*, vol. 26, no. 5, pp. 569–585, Oct. 2005.

[10] J. B. Blank *et al.*, "Overview of recruitment for the osteoporotic fractures in men study (MrOS)," *Contemp. Clin. Trials*, vol. 26, no. 5, pp. 557–568, Oct. 2005.

[11] S. R. Cummings *et al.*, "Appendicular bone density and age predict hip fracture in women. The Study of Osteoporotic Fractures Research Group," *JAMA*, vol. 263, no. 5, pp. 665–668, Feb. 1990.
